# Impaired PIEZO1 function drives uterine hypercontractility in adenomyosis-associated dysmenorrhea

**DOI:** 10.1093/hropen/hoag013

**Published:** 2026-02-17

**Authors:** Dingmin Yan, Yuedong Wang, Xishi Liu, Sun-Wei Guo

**Affiliations:** Department of Gynecology, Shanghai OB/GYN Hospital, Fudan University, Shanghai, China; Department of Statistics and Applied Probability, University of California, Santa Barbara, CA, USA; Department of Gynecology, Shanghai OB/GYN Hospital, Fudan University, Shanghai, China; Research Institute, Shanghai OB/GYN Hospital, Fudan University, Shanghai, China; Research Institute, Shanghai OB/GYN Hospital, Fudan University, Shanghai, China; Shanghai Key Laboratory of Female Reproductive Endocrine-Related Diseases, Fudan University, Shanghai, China; Shanghai Key Laboratory of Reproduction and Development, Fudan University, Shanghai, China

**Keywords:** adenomyosis, contractile amplitude, contractile irregularity, dysmenorrhea, contractility, endothelial nitric oxide synthase, nitric oxide, stiffness

## Abstract

**STUDY QUESTION:**

Does PIEZO1 play any role in adenomyosis-associated dysmenorrhea?

**SUMMARY ANSWER:**

PIEZO1 downregulation in the myometrium reduces the expression of endothelial nitric oxide synthase (eNOS) and nitric oxide (NO) production, leading to increased and irregular contractility that contributes to dysmenorrhea in women with adenomyosis.

**WHAT IS KNOWN ALREADY:**

Aberrant uterine contractility has long been documented in women with adenomyosis, but our knowledge of the molecular mechanisms governing uterine contractility is quite limited. Oxytocin receptor (OTR) expression in myometrium is elevated and correlates with uterine contractile amplitude and the severity of dysmenorrhea in women with adenomyosis. Adenomyosis induced in mice leads to increased uterine contractile amplitude and irregularity, accompanied by progressive generalized hyperalgesia. Additionally, increased myometrial vasopressin receptor (VP1αR) and potentially prostaglandin F2α (PGF_2α_) may also contribute to uterine hyperactivity.

**STUDY DESIGN, SIZE, AND DURATION:**

After written informed consent, we collected myometrial tissues ipsilateral and contralateral to adenomyotic lesions from 30 patients with adenomyosis (AM). As controls, normal myometrial tissue samples (CTL) were procured from 20 cycling women free of endometriosis and adenomyosis, age- and menstrual phase-matched with the AM group. Additionally, primary myometrial smooth muscle cells (SMCs) derived from 15 each AM and CTL subjects, age- and menstrual phase-matched, were cultured for gene and protein expression quantification and *in vitro* experimentation. In addition, 64 female Balb/C mice were randomly assigned in equal sizes to AM and CTL groups, with mice in the AM group receiving an AM induction procedure. Every 4-weeks post-induction until the 12th week, eight mice from each group were sacrificed, and their myometrial tissues were harvested. Uterine horn tissues were harvested and processed for histochemistry, immunohistochemistry (IHC), and Masson trichrome staining.

**PARTICIPANTS/MATERIALS, SETTING, AND METHODS:**

We performed Masson trichrome staining and IHC analysis of PIEZO1, PIEZO2, OTR, eNOS, phosphorylated eNOS (p-eNOS), and iNOS on myometrial tissue samples from AM and CTL groups. Western blotting analyses were performed to evaluate the protein expression levels of eNOS, p-eNOS, and iNOS in myometrial SMCs, and the concentration of NO metabolite was quantitated. Real-time RT-PCR and western-blotting analyses were performed to evaluate the gene and protein expression levels of PIEZO1, PIEZO2, and OTR under different substrate stiffness. The gene and protein expression levels of eNOS (and p-eNOS for protein) and iNOS were also evaluated after treatment of myometrial SMCs with PIEZO1 agonist Yoda1 and antagonist Dooku1, with or without non-selective NOS inhibitor L-NAME and selective iNOS inhibitor, 1400 W. The promoter methylation status at *PIEZO1* was evaluated by methylation-specific PCR (MSP) and *PIEZO1* expression levels after treatment with valproic acid (VPA) for 5 and 10 days were also evaluated. For mouse experiments, the extent of myometrial fibrosis was quantified via Masson trichrome staining. IHC analysis of Piezo1, Piezo2, Otr, eNos, p-eNos, and iNos on harvested myometrial tissue samples from AM and CTL groups was also performed. Bodyweight, hotplate latency, and uterine contractile amplitude and irregularity were measured.

**MAIN RESULTS AND THE ROLE OF CHANCE:**

Reduced PIEZO1 and eNOS staining and elevated PIEZO2 and OTR staining, concordant with the extent of fibrosis, were found in myometrium from women with adenomyosis, especially in myometrium proximal to adenomyotic lesions. No difference in iNOS staining was found between AM and CTL myometrium. Myometrial staining of OTR and PIEZO2 was positively correlated but the staining levels of PIEZO1, eNOS, and p-eNOS were negatively correlated with the dysmenorrhea severity. NO production was significantly reduced in AM myometrium as compared with controls. The gene and protein expression levels of PIEZO2 and OTR showed substrate stiffness-dependent increase while those of PIEZO1 showed stiffness-dependent decrease. Suppression and stimulation of PIEZO1 downregulated and upregulated eNOS expression as well as increased and reduced NO production, respectively. Consistent with the human data, mice with induced adenomyosis exhibited reduced Piezo1 and eNos staining and elevated Piezo2 and Otr staining in myometrium, concordant with increased fibrosis. Uterine contractile amplitude and irregularity were also increased progressively, and correlated with myometrial Piezo1/Piezo2 staining and hotplate latency. The *PIEZO1* promoter was hypermethylated in myometrium from women with adenomyosis, but the treatment with VPA reactivated *PIEZO1* expression.

**LARGE SCALE DATA:**

N/A.

**LIMITATIONS, REASONS FOR CAUTION:**

While we demonstrated stiffness-dependent reduction of PIEZO1 expression but an increase in PIEZO2 and OTR expression, the underlying mechanisms for these changes remain unelucidated. Further, although we provided evidence that eNOS expression and NO production are determined by PIEZO1 expression levels, the precise mechanisms were not fully elucidated.

**WIDER IMPLICATIONS OF THE FINDINGS:**

The stiffness-dependent increase in OTR and PIEZO2 expression, along with decreased PIEZO1 expression, underscores the progressive nature of adenomyosis. The link between PIEZO1 and eNOS, along with the discovery of *PIEZO1* hypermethylation and its downstream target eNOS, highlights the importance of mechanotransduction in adenomyosis and the role of NO in uterine contractility. While our study focused on adenomyosis-associated dysmenorrhea, the PIEZO1–eNOS aberration might also occur in other uterine disorders such as fibroids, and may also be a contributing factor for embryo implantation failure because of adenomyosis or other pathologies. Finally, given that VPA reactivates *PIEZO1* expression and subsequently induces eNOS, histone deacetylase inhibitors appear to be promising therapeutics, as has been shown in previous preclinical and pilot clinical studies.

**STUDY FUNDING/COMPETING INTERESTS:**

This research was supported in part by grant 82071623 (S.-W.G.) from the National Natural Science Foundation of China. S.-W.G. is a member of the Scientific Advisory Board of Heranova, BioSciences, E3A Healthcare, and of FimmCyte A.G., has provided consultancy advice to these companies, as well as to ReproNovo, but these activities had no bearing on this work. All other authors have no conflicts of interest.

WHAT DOES THIS MEAN FOR PATIENTS? Just as stomach spasms cause stomach ache, heightened and/or irregular uterine contractions are known to be responsible for dysmenorrhea (painful periods) in women with adenomyosis, a disease featuring the presence of uterine lining deep within the myometrium (muscle layers of uterus). We report that a gene called *PIEZO1* is suppressed in the myometrium in the vicinities of lesions from women with adenomyosis, and its suppression leads to reduced uterine production of nitric oxide—a natural substance that helps muscles relax. As a result, the uterus tends to have increased and spasmodic uterine contractions and thus dysmenorrhea. We also report that, when the muscle layer proximal to the adenomyotic lesions become heavily scarred and thus very stiff, *PIEZO1* is switched off by a chemical ‘silencing’ process, but it may be possible to turn it back on with certain medicines.

As of now, the treatment of adenomyosis mainly relies on hormonal drugs to cut off the estrogen supply or to stop menstruation. However, these drugs do not always work—especially when adenomyosis has caused extensive scarring in the uterus. Our findings suggest a novel, non-hormonal approach: easing the pain by suppressing uterine contractions directly. In the future, this could offer patients and their families a non-hormonal treatment option, with the goal of better pain relief and improved quality of life.

## Introduction

Adenomyosis is a common uterine disorder (Mishra *et al.*, 2023; Wang *et al.*, 2025) and a major cause for dysmenorrhea, pelvic pain, heavy menstrual bleeding (HMB), and subfertility (Farquhar and Brosens, 2006; Vercellini *et al.*, 2014; Harada *et al.*, 2016; Gordts *et al.*, 2018). Among these symptoms, adenomyosis-associated dysmenorrhea and pelvic pain are the most prevalent complaints and the most debilitating (Li *et al.*, 2014b).

Growing evidence indicates that hyperinnervation (Zhang *et al.*, 2010) and aberrant uterine contractility (Guo, 2022; Salmeri *et al.*, 2024), which lead to increased uterine pressure and hemodynamic dysfunction (Hellman *et al.*, 2018), contribute to pain generation. Studies have reported increased uterine contractile amplitude (Guo *et al.*, 2013; Rees *et al.*, 2024), along with decreased frequency (Guo *et al.*, 2013; Rees *et al.*, 2024) and velocity, as well as reduced contraction coordination (Rees *et al.*, 2024) in patients with symptomatic adenomyosis. It is conceivable that this heightened and irregular contractility, coupled with hyperinnervation and the activation of nociceptors (Nie *et al.*, 2010), causes dysmenorrhea.

However, our knowledge of the molecular mechanisms governing uterine contractility is quite limited. Elevated oxytocin receptor (OTR) expression has been observed in adenomyotic lesions, the junctional zone and the myometrium, which correlates with increased severity of dysmenorrhea and contractile amplitude (Mechsner *et al.*, 2010; Nie *et al.*, 2010; Guo *et al.*, 2013; Zhang *et al.*, 2015). In mouse models, induced adenomyosis led to augmented and irregular uterine contractility, accompanied by progressive generalized hyperalgesia (Mao *et al.*, 2011). In addition, increased myometrial vasopressin receptor (VP1αR) (Akerlund, 2004; Mechsner *et al.*, 2010) and potentially prostaglandin F2α (PGF_2α_) (Jusuf *et al.*, 2024) may also contribute to uterine hyperactivity. Derangements in potassium channels also have been implicated (Shi *et al.*, 2016).

While aberrant myometrial expression of OTR and VP1αR may be inotropic in adenomyosis, the role of nitric oxide (NO)—a potent smooth muscle relaxant and tocolytic agent (Norman, 1996; Grange *et al.*, 2001)—is less understood. As a signaling molecule involved in inflammation and oxidative stress (Forstermann and Sessa, 2012), NO is endogenously synthesized by the enzymatic action of NO synthase (NOS), which has three isoforms—neuronal (nNOS), inducible (iNOS), and endothelial (eNOS) (Forstermann and Sessa, 2012). In adenomyosis, one study reported significantly higher eNOS staining in the myometrium compared to controls (Oh *et al.*, 2013). In contrast, another study found higher staining and gene expression levels of iNOS, but *reduced* eNOS expression and comparable tissue nitrite concentration in the junctional zone (Liu *et al.*, 2013). Since eNOS must be phosphorylated (at Ser1117) to be activated and produce NO (Maccallini *et al.*, 2024), this discrepancy may stem from the fact that phosphorylated eNOS (p-eNOS) staining has not been systematically evaluated.

Adenomyotic lesions are fundamentally wounds undergoing repeated tissue injury and repair, ultimately resulting in fibrosis (Guo, 2018, 2022). This fibrogenic process in lesions can conceivably propagate into neighboring tissues, causing fibrosis along the way (Huang *et al.*, 2022; Mao *et al.*, 2023). As these tissues become more fibrotic and stiffer (Liu *et al.*, 2018), symptom severity—such as dysmenorrhea and HMB—exacerbates (Liu *et al.*, 2018; Huang *et al.*, 2022). Tissue stiffness is proportional to its resistance to deformation. The heightened tissue rigidity or stiffness alters the mechanobiology. For instance, YAP, the signaling molecule sensitive to rigidity of the extracellular matrix (ECM), is activated in the myometrium from patients with fibroids, concordant with increased collagen deposition (Purdy *et al.*, 2020). Not surprisingly, it is also activated in the junctional zone in adenomyosis (Huang *et al.*, 2021).

PIEZO1 and PIEZO2 are newly discovered mechanosensitive cation channels that transduce mechanical stress into Ca^2+^ dependent signals (Coste *et al.*, 2012). Mechanical activation of PIEZO channels generates a cationic non-selective current and channels that are permeable to Na^+^, K^+^, Ca^2+^, and Mg^2+^ (Parpaite and Coste, 2017). It is conceivable that the changing mechanical property resulting from lesional progression may involve these channels.

Following PIEZO1 activation, Ca^2+^ entry can lead to elevated NO production via eNOS activation by phosphoinositide 3-kinase/protein kinase A (PI3K/PKA) and PKB pathways (Zhang and Hintze, 2006) or through cAMP-mediated activation of PKA (Bir *et al.*, 2012). In the myometrium, 14-fold lower PIEZO1 expression is linked to preterm labor, suggesting higher myometrial PIEZO1 expression is associated with uterine quiescence while its downregulation is associated with heightened contraction (Barnett *et al.*, 2023), likely through NO release (Liu *et al.*, 2024). It can also induce eNOS in red blood cells via PKC (Sangha *et al.*, 2025). This raises the possibility that PIEZO1 downregulation in adenomyosis leads to reduced eNOS expression and NO production in myometrium, thereby augmenting uterine contractility, causing dysmenorrhea.

In contrast to PIEZO1, which is expressed broadly, PIEZO2 is expressed predominantly in somatosensory neurons (Szczot *et al.*, 2021). However, PIEZO2 has recently been identified as a key mechanoreceptor in pulmonary fibrosis (Freeberg *et al.*, 2025). Given the central role of fibrogenesis in lesional progression (Guo, 2022) and its connection with hyperinnervation (Zhang *et al.*, 2010), we hypothesized that in patients with adenomyosis, PIEZO2 is upregulated while PIEZO1 is downregulated, resulting in reduced eNOS expression and thus reduced NO production, which, in turn, may yield suppressed uterine relaxation and consequently increased contractility. This study was designed to test these hypotheses.

## Materials and methods

### Human samples

Uterine tissue samples were collected from patients diagnosed with adenomyosis (n = 30) and from control individuals without adenomyosis (n = 20) following the provision of informed consent. All participants were admitted to the Shanghai Obstetrics and Gynecology Hospital of Fudan University between August 2021 and January 2022. Adenomyosis diagnoses were established preoperatively via transvaginal ultrasound and pelvic MRI and were subsequently confirmed through postoperative histological examination.

After obtaining written informed consent, adenomyotic lesion samples (AM) were collected during hysterectomy procedures, promptly fixed in 10% buffered formalin, and processed for paraffin embedding. For the control group (CTL), endometrial and myometrial tissue samples were obtained from women undergoing hysterectomy due to cervical high-grade squamous intraepithelial lesion, who had no clinical evidence or history of adenomyosis, endometriosis, or uterine fibroids. The control subjects were frequency-matched to the adenomyosis patients based on age and menstrual phase.

None of the patients had received hormonal or anti-platelet therapies for at least 3 months prior to surgery, and individuals with malignant or inflammatory conditions were excluded. All tissue samples were collected following the acquisition of comprehensive written informed consent. A flow chart illustrating the inclusion and exclusion criteria is shown in Supplementary Fig. S1. This study was approved by the Institutional Ethics Review Board of the Shanghai Obstetrics and Gynecology Hospital (Approval ID: 2021-35; updated 2024-115).

### Immunohistochemistry

Technical procedural details are provided in the Supplementary Materials and Methods.

### Masson trichrome staining

Masson trichrome staining was employed to evaluate collagen fiber deposition within myometrial tissue specimens. Tissue sections were deparaffinized in xylene and rehydrated through a graded alcohol series. Subsequently, the sections were immersed in Bouin’s solution, prepared with 75 ml of saturated picric acid, 25 ml of 10% (w/v) formalin solution, and 5 ml of acetic acid, and incubated at 37 °C for 2 h. Staining was performed using a commercial Masson’s Trichrome Staining kit (Baso, Wuhan, China) in accordance with the manufacturer’s protocol. The relative area of blue-stained collagen fibers within the myometrium was quantified using Image Pro-Plus software (Version 6.0, Media Cybernetics, Inc., Rockville, MD, USA) and expressed as a proportion of the total field area. All histological and histochemical scorings were performed by a single investigator (D.Y.), who was completely blinded to the group identity of the slides being evaluated.

### Animals

A total of 64 6-week-old virgin female Balb/C mice were acquired from the SLAC Experimental Animal Company (Shanghai, China) for use in this study. The animals were maintained under controlled conditions, including a 12-h/12-h light–dark cycle, with *ad libitum* access to food and water. All experiments were conducted in accordance with the guidelines outlined in the National Research Council’s Guide for the Care and Use of Laboratory Animals (National Research Council, 2011) and were approved by the Institutional Experimental Animals Review Board of Shanghai Obstetrics and Gynecology Hospital, Fudan University.

### Induction of adenomyosis

The 64 mice were randomly assigned in equal sizes to the AM group and the control (CTL) group. Adenomyosis was induced using an established mouse model involving endometrial–myometrial interface (EMI) disruption (EMID), as previously described (Hao *et al.*, 2020). In brief, following anesthesia with 2% chloral hydrate (w/v), a 2-cm midline abdominal incision was made to expose the uterine horns. A microcatheter and guidewire (STD125-26S; Asahi Intecc, Tambol Bangkadi, Thailand), typically employed for uterine artery embolization, were inserted into the murine uterine horns through the vaginal canal. The guidewire tip was gently scraped along the EMI of each uterine horn in a top-to-bottom manner. Twelve weeks after the procedure, adenomyosis was successfully induced in all mice in the AM group (Hao *et al.*, 2020). Control mice underwent the same surgical incision as the experimental group but did not receive the EMID procedure.

Every 4-weeks post-induction until the 12th week, eight mice from each group were sacrificed. Bodyweight, hotplate latency, and uterine contractile amplitude and frequency were measured at 4-week intervals until the time of sacrifice. For behavioral assessments, two independent observers performed the evaluations, with one of the observers being unaware of the identity of the group assignment. Uterine horn tissues were harvested and processed for histochemistry, immunohistochemistry (IHC), Masson trichrome staining, as well as for contractility evaluation.

### Cell culture and reagents

Primary cultures of human uterine smooth muscle cells (uHSMCs) were established from the biopsy according to the explant technique, as previously described by Streuli *et al.* (2015). Within 2 h following surgical excision, uterine smooth muscle biopsies were dissected into fragments not exceeding 5 mm in dimension. The fragments were distributed into a 6-well plate (Corning, New York, NY, USA) and covered with a glass slide. Two drops of DMEM-high glucose (Cytiva, HyClone, Logan, UT, USA), supplemented with 20% fetal bovine serum (FBS) (Gibco Laboratories, Life Technologies, Grand Island, NY, USA), 100 mmol/l HEPES, and antibiotics (100 IU/ml penicillin, 100 mg/ml streptomycin, and 2.5 μg/ml Amphotericin B, HyClone), were added around the fragment. After 48 h of incubation, 1 ml of complete culture medium containing 20% FBS was added. The culture medium was refreshed twice per week. After approximately 15 days, when the fragments were surrounded by outgrown smooth muscle cells (SMCs), the explants were removed. At around 21 days of culture, upon reaching ∼90% confluency, the cells were treated with trypsin and subcultured into 25-cm^2^ cell culture flasks (Corning, New York, NY, USA) in DMEM supplemented with 10% FBS. For all experiments, cells between the passages 2–5 were used. The purity and identity of the cell cultures were confirmed through routine immunocytochemical analysis for α-smooth muscle actin (α-SMA) staining.

Cells with different treatments were used for quantitative RT-PCR, western-blot analysis, and NO content determination. The small molecules Yoda1 (MedChemExpress, Monmouth Junction, NJ, USA), a selective PIEZO1 agonist, and Dooku1 (MedChemExpress), a selective PIEZO1 antagonist, were employed to enable precise experimental modulation of PIEZO1 channel activity (Evans *et al.*, 2018; Botello-Smith *et al.*, 2019). Stock solutions of both compounds were dissolved in dimethyl sulfoxide and diluted in appropriate buffers immediately prior to use. Additionally, N^w^-nitro-L-arginine methyl ester (L-NAME) (MedChemExpress), a non-selective inhibitor of NO synthase (NOS), and N-(3-(aminomethyl) benzyl) acetamidine (1400 W) (MedChemExpress), a selective inhibitor of inducible NOS (iNOS) (Oliveira-Neto *et al.*, 2024), were dissolved in H_2_O to prepare stock solutions, which were subsequently diluted in appropriate buffers before application in experiments.

### Preparation of polyacrylamide gels

Polyacrylamide gel substrate (PGS) is a widely used material in the investigation of cellular behavior due to its excellent biocompatibility and adjustable mechanical properties (Kandow *et al.*, 2007). We prepared polyacrylamide gels with target stiffness values of 5, 30, and 50 kPa to mimic different tissue stiffness, as described previously (Huang *et al.*, 2022). The specific experimental procedures can be found in the Supplementary Materials and Methods.

### RNA isolation and real-time RT-PCR and western-blot analysis

Total RNA was isolated from uHSMCs that had been cultured for 72 h on PGS with stiffness values of 5 kPa, 30 kPa, and 50 kPa. After washing with phosphate-buffered saline (HyClone), uHSMCs were directly reverse-transcribed into cDNA using an EZ‐press Cell to cDNA Kit (B0003C, EZ Bioscience, Roseville, CA, USA) according to the manufacturer’s instructions. The mRNA abundance was assessed using SYBR Green qPCR Master Mix (A0001‐R, EZ Bioscience). Expression levels were normalized to the geometric mean of *GAPDH* values, and the quantification of mRNA abundance was performed using the method as described previously (Livak and Schmittgen, 2001). The gene names and corresponding primers sequences used in this study are provided in Table 1.

**Table 1.** List of primers used in the real-time RT-PCR and methylation-specific PCR analysis.

Gene name	Sequence	
*GAPDH*	forward	5′-GCACCGTCAAGGCTGAGAAC-3′
	reverse	5′-TGGTGAAGACGCCAGTGGA-3′
*OTR*	forward	5′-GTGGTGGCAGTGTTTCAGGT-3′
	reverse	5′-CGTAGAAGCGGAAGGTGATG-3′
*PIEZO1*	forward	5′-CGTCACCGTCATCATCTCCAAG-3′
	reverse	5′-ACAGGCAGCAGGCAGTCC-3′
*PIEZO2*	forward	5′-CACCTGGCTACAACTGCTCAAC-3′
	reverse	5′-GTTACTCTGTGCTGCTTCGTCTG-3′
*ENOS*	forward	5′-TGATGGCGAAGCGAGTGAAG-3′
	reverse	5′-ACTCATCCATACACAGGACCC-3′
*INOS*	forward	5′-AAGGAGCGGCACAGTATGAAA-3′
	reverse	5′-TCACCACTGATAATGACGCAC-3′
*ME-PIEZO1*	forward	5′-AGGGTAGACGAAGGTTAAATTGAC-3′
	reverse	5′-AAACGATAAAAATAAAAACCACGAC-3′
*UM-PIEZO1*	forward	5′-AAGGGTAGATGAAGGTTAAATTGAT-3′
	reverse	5′-AAACAATAAAAATAAAAACCACAAC-3′

Procedures for performing western-blot analysis are detailed in the Supplementary Materials and Methods.

### NO content determination

Homogenized tissue samples were prepared using the method described in the preceding section. For agonist and antagonist experiments, uHSMCs were seeded at a density of 1 × 10^5^ cells per well in 6-well plates with 3 ml of culture medium and incubated at 37 °C under 5% CO_2_ for 24 h. The culture medium was then replaced, and Yoda1 and Dooku1 were administered at a final concentration of 3 µM (Barnett *et al.*, 2023) and 10 µM (Matsunaga *et al.*, 2021; Hwang *et al.*, 2024), respectively. A subset of the cells was pretreated with L-NAME (100 µM) (Porto Ribeiro *et al.*, 2022) and 1400 W (10 µM) (Passos *et al.*, 2023) for 30 min prior to the addition of Yoda1 (3 µM). The control well received vehicle only. The plates were subsequently returned to the 37 °C, 5% CO_2_ incubator for 48 h. NO contents in cell lysates was determined using a Micro NO Content Assay Kit (Solarbio, Shanghai, China) in accordance with the manufacturer’s instructions. The optical density was measured at a wavelength of 550 nm using a Bioteck microplate reader.

### Methylation-specific PCR

Cell fractions were isolated from myometrial tissue samples by microdissection. Genomic DNA was extracted using microDNA extraction kits (DP316, Tiangen, Beijing, China), treated with heavy sulfite (DP215-02, Tiangen), and subsequently amplified by PCR using specifically designed methylation (ME) and non-methylation (UM) primers (EM101-01, Tiangen). The PCR-amplified products were subjected to agarose gel electrophoresis. The primer sequences used for methylation-specific PCR (MSP) are listed in Table 1.

Primers were designed using MethPrimer (http://www.urogene.org/cgi-bin/methprimer/methprimer.cgi).

### Measuring uterine contractile irregularity using wavelets

The uterine contractility data are signals recorded over time and can be viewed as time series. For regular contractions, contractility can be described in terms of frequency and magnitude. However, in adenomyotic uteri, contractions are highly irregular, making frequency measures less meaningful. A powerful approach for analyzing such data is wavelet analysis, which enables signal representation that facilitates the extraction and characterization of key features otherwise hidden in the data.

We applied wavelet decomposition to assess contractile irregularity (Mao *et al.*, 2011). This method decomposes the signal into components that capture variations across different frequencies and times. High-frequency components, reflecting rapid fluctuations, were used to quantify irregularity by summing the squared wavelet coefficients after filtering out slow trends. For each mouse, the 1024-point signal was analyzed using Daubechies’s Least Asymmetric Wavelet filter of length 10 with the *wavethresh* package in R. This yielded 1023 coefficients across nine resolution levels. Irregularity was defined as the sum of squares of the 768 coefficients from the top two levels (512 + 256).

### Statistical analysis

Fisher’s exact test was used for contingency table analysis. The comparison of distributions of continuous variables between two groups was made using Wilcoxon’s test. Pearson’s correlation coefficient was calculated when analyzing the correlation between two continuous variables, but Spearman’s correlation coefficient was used when one or both variables were ordinal. Multiple linear regression was used to identify factors associated with the immunostaining levels. It was also used to see whether there is a duration-dependent effect on the gene expression levels of *PIEZO1*.

P-values of <0.05 were considered statistically significant. All computations were made with R (Version 4.5.1., Vienna, Austria) (R Core Team, 2016).

## Results

### Reduced PIEZO1 and eNOS staining and elevated PIEZO2 staining in myometrium concordant with the extent of fibrosis

To see whether PIEZO channels play any role in adenomyosis, we first performed IHC analysis of PIEZO1, PIEZO2, OTR, eNOS, p-eNOS, and iNOS in both control myometrium and myometrium neighboring (ipsilateral) and away from (contralateral) adenomyotic lesions, and quantitated the extent of myometrial fibrosis by Masson trichrome staining. To do this, we collected the myometrial tissue ipsilateral to the lesions based on the result of MRI and the stiffest myometrial areas by palpation after hysterectomy.

We also procured contralateral myometrial tissues as myometrial regions, per MRI, that are distal to the lesions—specifically the lower uterine segment for fundal lesions, the posterior wall for anterior wall lesions, or vice versa—along with the palpated soft tissues. The characteristics of all recruited subjects are listed in Table 2.

**Table 2.** Characteristics of recruited patients who donated their tissue samples for this study.

Variable	Controls (n = 20)	Adenomyosis (n = 30)	Statistical significance
Age (in years)			0.706
* Mean±SD*	39.2 ± 5.1	39.9 ± 4.3	
* Median (Range)*	40 (27–46)	39 (32–48)	
Menstrual phase			0.327
* Proliferative*	8 (40.0%)	8 (26.7%)	
* Secretory*	12 (60.0%)	22 (73.3%)	
Parity			0.013
* 0*	1 (5.0%)	4 (13.3%)	
* 1*	8 (40.0%)	20 (66.7%)	
* 2*	9 (45.0%)	5 (16.7%)	
* ≥3*	2 (10.0%)	1 (3.3%)	
Uterus size (cm^3^)	NA		NA
* Mean±SD*		249.5 ± 156.9	
* Median (Range)*		202.2 (77.4–828.8)	
Severity of dysmenorrhea			6.82 × 10^−10^
* None*	20 (100.0%)	0 (0.0%)	
* Mild*	0 (0.0%)	1 (3.3%)	
* Moderate*	0 (0.0%)	4 (13.3%)	
* Severe*	0 (0.0%)	25 (83.3%)	
Kishi’s classification of adenomyosis	NA	30 (100.0%)	NA
* Intrinsic*		14 (46.7%)	
* Extrinsic*		9 (30.0%)	
* Intramural*		0 (0.0%)	
* Indeterminate*		7 (23.3%)	
Co-occurrence with endometriosis			0.007
* No*	20 (100.0%)	21 (70.0%)	
* Yes*	0 (0.0%)	9 (30.0%)	
Co-occurrence with myoma^#^			0.092
* No*	20 (100.0%)	26 (86.7%)	
* Yes*	0 (0.0%)	4 (13.3%)	

# Co-occurrence with exclusively FIGO Type 6 and < 3 cm in diameter, and Type 7 myoma.

For continuous data, Wilcoxon’s rank test was used. For categorical data, Fisher’s exact test was used. NA, not applicable.

**Figure 1.**
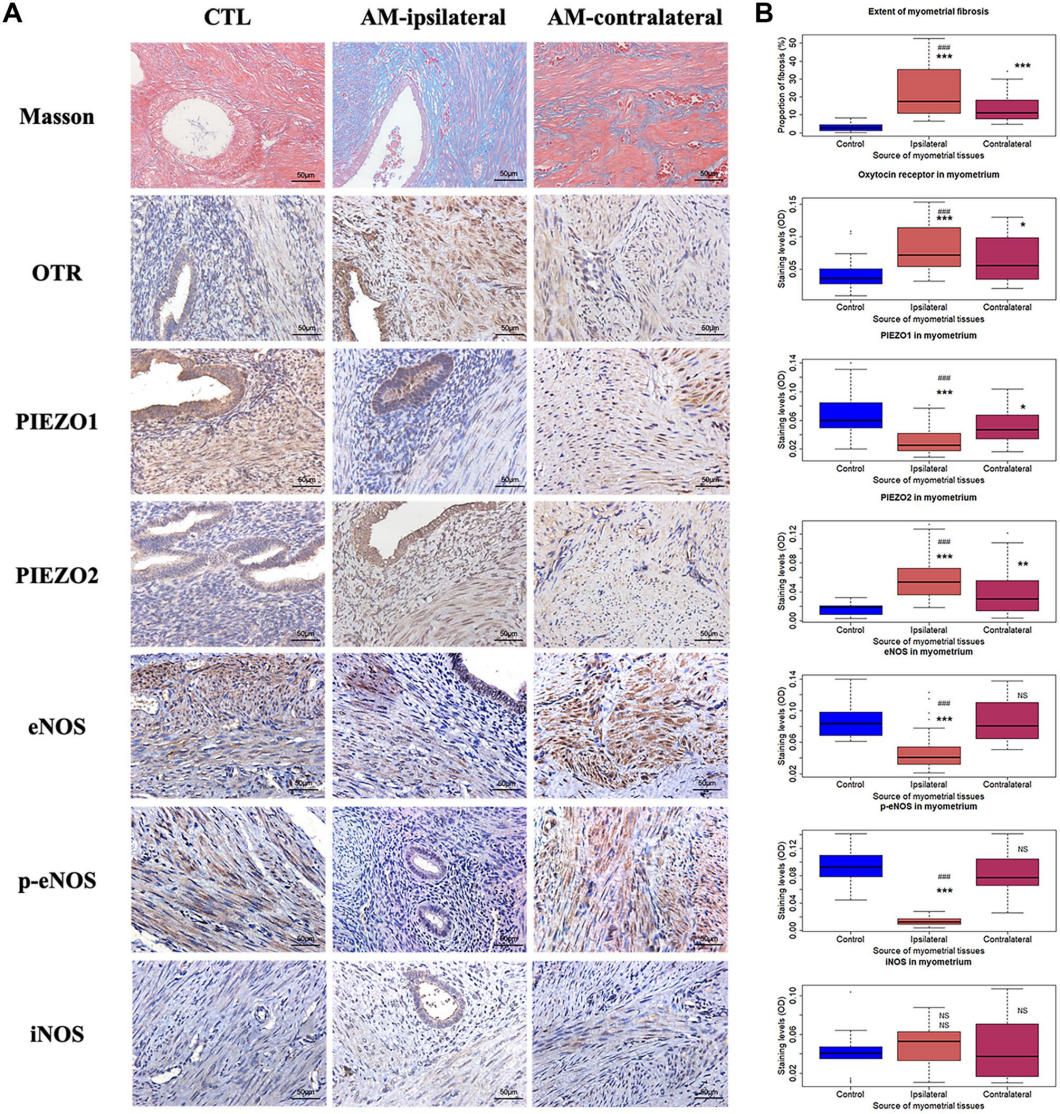
**Results of histochemistry and immunohistochemistry analyses of myometrial tissue samples.** The left panel exhibits representative photomicrographs of the extent of fibrosis via Masson trichrome staining and immunostaining of OTR, PIEZO1, PIEZO2, eNOS, p-eNOS, and iNOS in both control myometrium and myometrium neighboring (AM-ipsilateral) and away from (AM-contralateral) adenomyotic lesions. The right panel shows the data summary for each histochemistry and immunohistochemistry marker. Sample sizes: CTL: n = 20; AM: n = 30. Magnification: × 400. Scale bar, 50 μm. Symbols for statistical significance levels: NS: *P* > 0.05; *: *P* < 0.05; **: *P* < 0.01; *** and ###: *P* < 0.001 (by Wilcoxon’s rank test, *: compared with the CTL group; # in comparison with the contralateral myometrium). CTL, control myometrium; AM, adenomyosis; OTR, oxytocin receptor; eNOS, endothelial nitric oxide synthase; p-eNOS, phospho-endothelial nitric oxide synthase; iNOS, inducible nitric oxide synthase.

We found that PIEZO1, PIEZO2, and OTR staining was seen predominantly in the membrane of epithelial and myometrial cells (Fig. 1A). The immunoreactivity against eNOS, p-eNOS, and iNOS was seen mostly in membrane and cytoplasm of myometrial cells and was also detected in the endothelium (Fig. 1A).

As expected, the extent of myometrial fibrosis of both ipsilateral and contralateral to lesions was significantly higher than that of control myometrium (both *P*-values ≤1.2×10^−9^; Fig. 1B). In uterine myometrial cells both surrounding and distal to the lesions, the immunoexpression of OTR and PIEZO2 was significantly higher than that of control myometrium. In contrast, the PIEZO1 staining levels in both ipsilateral and contralateral myometrium were significantly lower than that of controls (all 6 *P*-values ≤ 0.042; Fig. 1B). Multiple linear regression incorporating age, menstrual phase, parity, co-occurrence with endometriosis and/or uterine fibroids, and group identity (AM vs CTL) also confirmed the above results (all *P*-values ≤ 5.4 × 10^−5^; all *R*^2^’s ≥ 0.29).

The immunoexpression of both eNOS and p-eNOS in the myometrium ipsilateral to the adenomyotic lesion was significantly lower than that in the contralateral and control myometria, especially p-eNOS (all 4 *P*-values ≤ 4.2 × 10^−4^; Fig. 1B). In contrast, their immunoexpression in contralateral myometrium was comparable to that in the normal myometrium (both *P*-values ≥ 0.11; Fig. 1B). Multiple linear regression incorporating age, menstrual phase, parity, co-occurrence with endometriosis and/or uterine fibroids, and group identity also confirmed the above results (all *P*-values ≤ 1.2 × 10^−6^; all *R*^2^’s ≥ 0.39). For iNOS, no statistically significant difference in its immunoexpression between the control and ipsilateral or lateral myometria was found (both *P*-values ≥ 0.19; Fig. 1B).

The eNOS staining in ipsilateral and contralateral myometria as well as control myometrium correlated positively with that of p-eNOS (*r* = 0.72, *P* = 4.6 × 10^−14^). Both eNOS and p-eNOS staining levels were positively correlated with that of PIEZO1 (both *r*’s ≥ 0.56, both *P*-values ≤ 5.7 × 10^−8^). These correlations were still highly significant within the AM group (all 3 *r*’s ≥ 0.55, all 3 *P*-values ≤ 5.0 × 10^−6^). The immunoreactivity against OTR, PIEZO1, PIEZO2, eNOS, p-eNOS, and iNOS, as well as the extent of fibrosis, between the ipsilateral and contralateral myometria was positively correlated (all 7 *r*’s ≥ 0.52, all 7 *P*-values ≤ 0.003).

The immunostaining of PIEZO1 and PIEZO2 was negatively correlated (*r*= −0.77, *P* < 2.2 × 10^−16^; Supplementary Fig. S2). OTR staining levels correlated negatively with that of PIEZO1 (*r*=−0.82, *P* < 2.2 × 10^−16^; Supplementary Fig. S2) but positively with that of PIEZO2 (*r* = 0.80, *P* < 2.2 × 10^−16^; Supplementary Fig. S2). Similarly, the staining levels of p-eNOS correlated positively with that of PIEZO1 (*r* = 0.63, *P* = 2.7 × 10^−10^; Supplementary Fig. S2) but negatively with that of PIEZO2 (*r*=−0.60, *P* = 5.1 × 10^−9^; Supplementary Fig. S2). The staining levels of p-eNOS correlated negatively with that of OTR (*r* = −0.46, *P* = 1.6 × 10^−5^; Supplementary Fig. S2). The extent of fibrosis correlated positively with the staining levels of both OTR and PIEZO2 (both *r*’s ≥ 0.85, both *P*-values < 2.2 × 10^−16^; Supplementary Fig. S2) but negatively with the staining levels of both PIEZO1 and p-eNOS (both *r*’s ≤ −0.60, both *P*-values ≤ 3.9 × 10^−9^; Supplementary Fig. S2).

**Figure 2.**
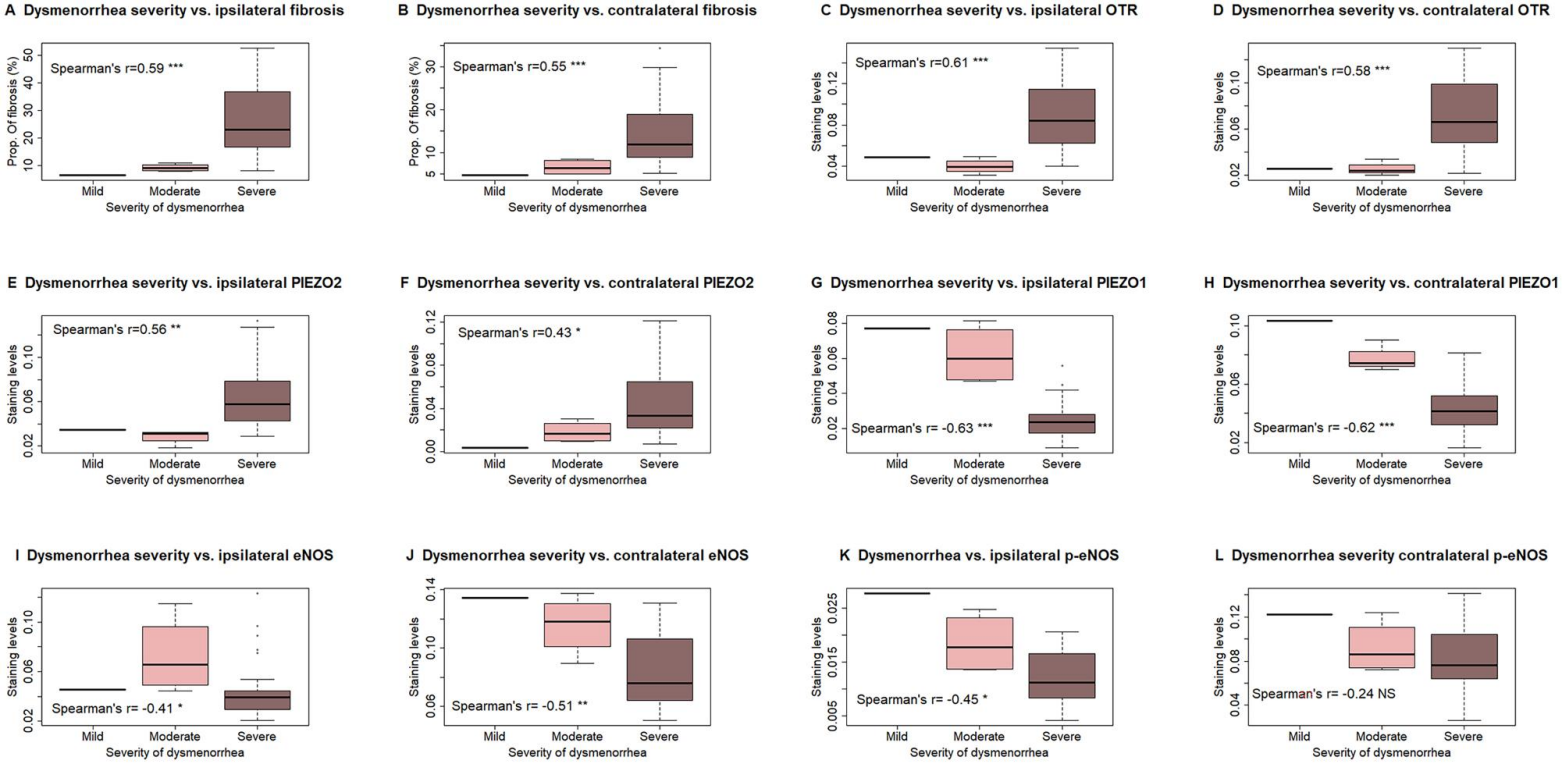
**Relationship between the severity of dysmenorrhea and histochemistry and immunohistochemistry markers.** Boxplots showing the relationship between the severity of dysmenorrhea and the extent of ipsilateral myometrium (**A**), contralateral myometrium (**B**), OTR staining levels in ipsilateral myometrium (**C**), OTR staining levels in contralateral myometrium (**D**), PIEZO2 staining levels in ipsilateral myometrium (**E**), PIEZO2 staining levels in contralateral myometrium (**F**), PIEZO1 staining levels in ipsilateral myometrium (**G**), PIEZO1 staining levels in contralateral myometrium (**H**), eNOS staining levels in ipsilateral myometrium (**I**), eNOS staining levels in contralateral myometrium (**J**), iNOS staining levels in ipsilateral myometrium (**K**), and p-eNOS staining levels in contralateral myometrium (**L**). The Spearman’s correlation coefficient is shown in each plot, with its statistical significance level. Symbols for statistical significance levels: NS: *P* > 0.05; *: *P* < 0.05; **: *P* < 0.01; ***: *P* < 0.001.

By combining those with a dysmenorrhea visual analog scale score between 0–3 as mild, 4–6 as moderate, and over 6 as severe, we found that, within patients with adenomyosis, the extent of both ipsilateral and contralateral myometrial fibrosis was positively correlated with the severity of dysmenorrhea (Spearman’s *r* ≥ 0.59, *P* ≤ 0.0017; Fig. 2A and B). The ipsilateral and contralateral myometrial staining of OTR, PIEZO2 were also positively correlated with severity of dysmenorrhea (all 4 Spearman’s *r*’s ≥ 0.43, *P*’s ≤ 0.018; Fig. 2C–F). With exception of contralateral p-eNOS staining (*r*= −0.24, *P* = 0.20; Fig. 2L), the ipsilateral and contralateral staining levels of PIEZO1, eNOS, and p-eNOS were all negatively correlated with the dysmenorrhea severity (all 5 Spearman’s *r*’s ≤ −0.41, *P* ≤ 0.025; Fig. 2G−K).

### Reduced NO content in the ipsilateral myometrium concordant with reduced PIEZO1 and eNOS expression

**Figure 3.**
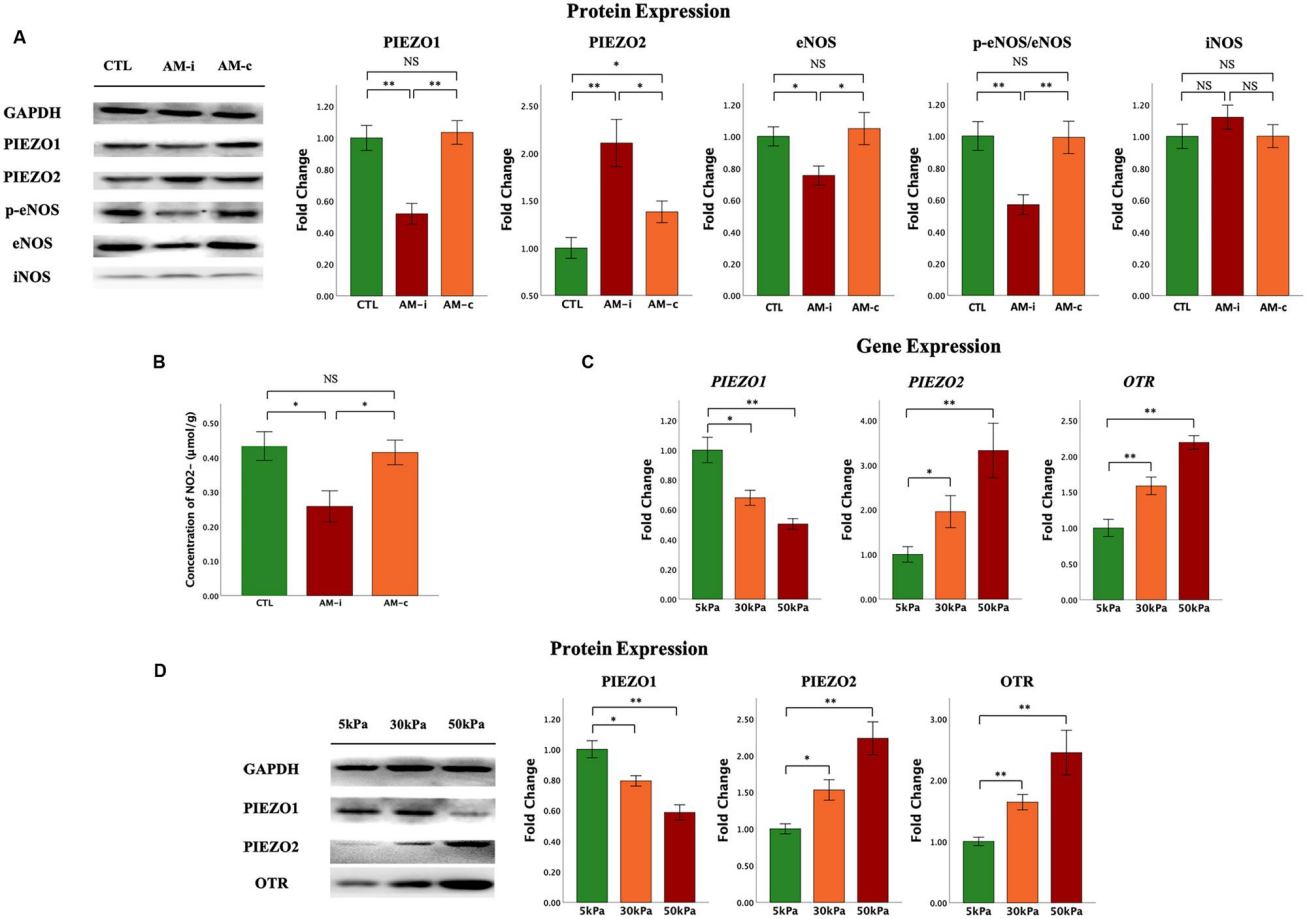
**The expression of PIEZO1, PIEZO2, p-eNOS, eNOS, and the NO content in the myometrium of adenomyosis, and the effect of matrix stiffness on PIEZO1 and PIEZO2 expression in human uterine smooth muscle cells (uHSMCs).** (**A**) The protein expression of PIEZO1, eNOS, and p-eNOS in the ipsilateral myometrium of adenomyotic lesions was significantly lower than that in the contralateral myometrium and normal myometrium, especially the change of p-eNOS. In both ipsilateral and contralateral myometrium from AM patients, the expression of PIEZO2 was significantly higher than that of CTL. All values were normalized to GAPDH expression. (**B**) The nitrite content in the ipsilateral myometrium around the lesions was significantly lower than that in the contralateral and normal myometrium. Real-time PCR and western-blot analysis showed the gene (**C**) and protein (**D**) expression of PIEZO1, PIEZO2, and OTR in uHSMCs cultured on different stiffness of polyacrylamide gels for 72 h. Expression normalized to GAPDH (n = 5). Data are represented in means ± SDs, and the comparison is shown as indicated by Wilcoxon’s rank test. N = 5 in each group. Symbols for statistical significance levels: NS: *P* > 0.05; *: *P* < 0.05; **: *P* < 0.01; ***: *P* < 0.001. AM, adenomyosis; CTL, control myometrium; AM-i (AM-ipsilateral), myometrium neighboring adenomyotic lesions; AM-c (AM-contralateral), myometrium away from adenomyotic lesions.

Next, we validated the protein expression levels of PIEZO1, PIEZO2, eNOS, and iNOS in myometrium ipsilateral and contralateral to adenomyotic lesions. Consistent with the IHC data, we found that the expression levels of PIEZO1, eNOS, and p-eNOS in the ipsilateral myometrium were significantly lower than those in the contralateral and normal myometria, especially p-eNOS (both *P*-values ≤ 0.028; Fig. 3A), but no significant difference was found between the contralateral and normal myometria (both *P*-values ≥ 0.60). In both ipsilateral and contralateral myometria, the expression levels of PIEZO2 were significantly higher than that of control (both *P*-values ≤ 0.032; Fig. 3A), while their expression levels in the ipsilateral myometrium remained significantly higher than in its contralateral counterpart (*P* = 0.032; Fig. 3A). The expression levels of iNOS in SMCs derived from the ipsilateral myometrium were slightly higher than those of control SMCs but the difference did not reach statistical significance (*P* = 0.22; Fig. 3A). No significant difference in iNOS protein levels was found between contralateral myometrial and control SMCs (*P* = 1.00; Fig. 3A). These results are consistent with the IHC data.

We also evaluated the content of NO metabolites in the myometrium of AM patients. The nitrite content in myometrium ipsilateral to the lesion was significantly lower than that in the contralateral and normal myometria (both *P*-values = 0.032; Fig. 3B), while no difference between the contralateral and control myometria was found (*P* = 1.00). Thus, the myometrial expression of eNOS/p-eNOS, but not iNOS, is associated with and likely to determine the NO content in myometrium, which, in turn, determines the uterine contractility in AM patients.

### Matrix stiffness-dependent gene and protein expression of PIEZO1 and PIEZO2 in human uHSMCs

Given the close association between the extent of myometrial fibrosis and the immunoexpression and protein expression of PIEZO1, PIEZO2, and OTR as shown above, we hypothesized that increased ECM stiffness would determine the expression of PIEZO1, PIEZO2, and possibly OTR in human myometrial SMC cells. To test this hypothesis, we cultured uHSMCs on compliant (5-kPa PGS), moderate (30-kPa PGS), and rigid (50-kPa PGS) substrates for 3 days. By real-time PCR assays, we found that the expression levels of *PIEZO1* in uHSMCs grown on moderate (30 kPa) and rigid substrates (50 kPa) were all significantly reduced as compared with those grown on soft substrates, while that of *PIEZO2* and *OTR* was significantly elevated (all *P*-values ≤ 0.027; Fig. 3C). Consistent with the gene expression, cells grown on moderate (30 kPa) and rigid substrates (50 kPa) had significantly lower protein expression levels of PIEZO1 than those grown on soft substrates (5 kPa), while that of PIEZO2 and OTR was significantly elevated as the matrix stiffness increased (all *P*-values ≤ 0.0021; Fig. 3D).

Thus, while myometrial expression of PIEZO2 and OTR increases as the myometrium becomes more fibrotic and stiffer, the PIEZO1 expression decreases.

### Antagonism and stimulation of PIEZO1 downregulates and upregulates eNOS expression and increases and reduces NO production, respectively

**Figure 4.**
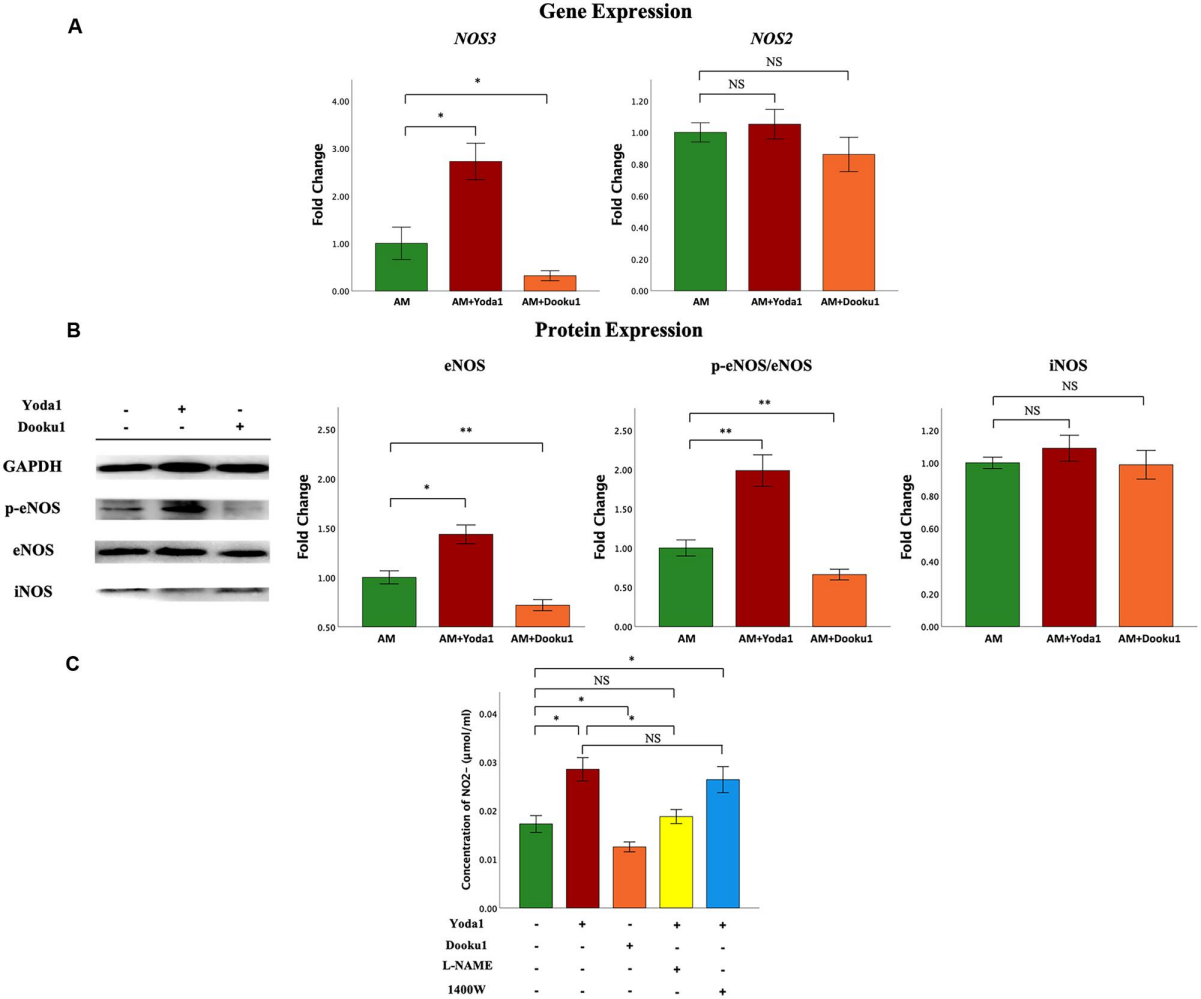
**The effects of PIEZO1 agonists and inhibitors on the expression of eNOS/p-eNOS and iNOS, as well as their impact on NO production.** After treatment with Yoda1 (3 μM) or Dooku1 (10 μM) for 72 h, (**A**) the gene expression level of *NOS3* (coding for eNOS) and *NOS2* (coding for iNOS), (**B**) and the protein expression level of p-eNOS/eNOS and iNOS in uHSMCs as compared with control. Data are represented in mean±SD, and the comparison is shown as indicated by Wilcoxon’s rank test. (**C**) The nitrite content in uHSMCs after treatment with Yoda1 (3 μM), Dooku1 (10 μM), non-selective NOS inhibitor L-NAME (100 µM), and 1400 W (10 µM) (a selective inhibitor for iNOS). In all experiments, n = 5 in each group. Symbols for statistical significance levels: NS: *P* > 0.05; *: *P* < 0.05; **: *P* < 0.01. CTL, control myometrium; AM-i (AM-ipsilateral), myometrium neighboring adenomyotic lesions; AM-c (AM-contralateral), myometrium away from adenomyotic lesions.

To further investigate whether PIEZO1 regulates eNOS and subsequent NO production in myometrium, we treated uHSMCs with Yoda1 (3 μM), a PIEZO1 agonist, and Dooku1 (10 μM), a PIEZO1 antagonist, for 72 h. We found that the gene expression level of *NOS3* (coding for eNOS) was significantly elevated after treatment with Yoda1 as compared with those treated with vehicle (*P* = 0.016). In contrast, treatment with Dooku1 significantly reduced *NOS3* expression (*P* = 0.032; Fig. 4A). The treatment had no impact on the expression of *NOS2* (coding for iNOS; both *P*-values ≥ 0.31; Fig. 4A).

Consistent with the gene expression, Yoda1 treatment significantly increased the protein expression levels of eNOS and p-eNOS, especially p-eNOS (both *P*-values ≤ 0.028), while Dooku1 treatment significantly reduced expression levels of eNOS and p-eNOS (both *P*-values ≤ 0.009; Fig. 4B). However, PIEZO1 suppression or activation did not have any effect on iNOS expression (both *P*-values ≥ 0.47; Fig. 4B), confirming that *NOS3* is a downstream gene of PIEZO1.

Next, we measured the content of NO products in uHSMCs. After PIEZO1 activation by Yoda1, the nitrite content was elevated significantly as compared with the control (*P* = 0.016). In contrast, the nitrite content decreased significantly after PIEZO1 suppression by Dooku1 (*P* = 0.032; Fig. 4B), establishing the link between PIEZO1 expression and myometrial NO production.

To see which NOS is responsible for the NO production, we used its inhibitors. However, since there is no highly selective inhibitor for eNOS, we used the non-selective NOS inhibitor L-NAME but for iNOS, its selective inhibitor, 1400 W. After pretreatment with L-NAME (100 μM) for 30 min and then with Yoda1 for 72 h, the nitrite content in uHSMCs was comparable to the control (*P* = 0.42), but was significantly lower than that of Yoda1 treatment alone (*P* = 0.016). However, the nitrite content in uHSMCs still increased significantly (*P* = 0.032) after pretreatment with 1400 W (10 μM) for 30 min and then with Yoda1 for 72 h, and there was no statistical difference compared with the Yoda1 treatment alone (*P* = 0.69; Fig. 4C). Taken together, these results indicate that PIEZO1 induction increases the NO production in uHSMCs through eNOS phosphorylation, increases the synthesis of NO, and thus mediates the relaxation of uterine smooth muscles.

### Uterine contractility correlates with myometrial Piezo1/Piezo2 expression levels in mice with induced adenomyosis

To see whether adenomyosis can result in increased PIEZO2 and OTR expression and reduced expression of PIEZO1 and eNOS as well as increased uterine contractility, we carried out a serial mouse experiment using the EMID model (Hao *et al.*, 2020). We evaluated uterine contractility and irregularity, along with the myometrial immunoreactivity against Otr, Piezo1, Piezo2, eNos, and p-eNos at 0, 4, 8, and 12 weeks after induction of adenomyosis, with the purpose to see how these markers change over the entire adenomyosis development period as by week 12, adenomyosis was fully established in all mice (Hao *et al.*, 2020). No mouse died during the experiment, and no adverse effect was found. Adenomyosis was successfully induced in all mice per histological confirmation by H&E staining.

**Figure 5.**
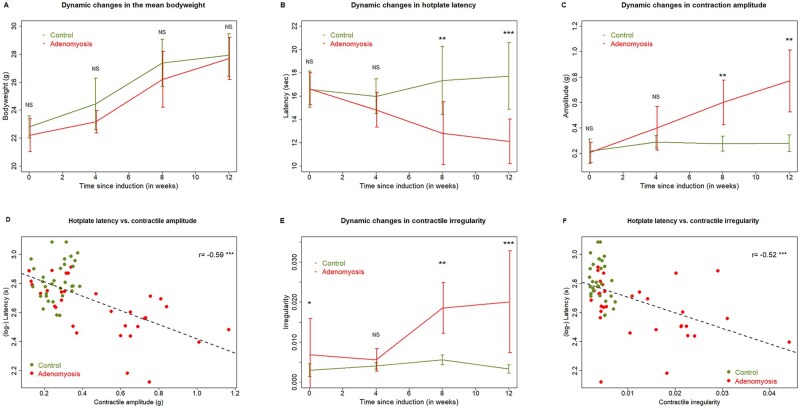
**Summary of mouse experiment data.** Dynamic changes in mean bodyweight (**A**), hotplate latency (**B**), uterine contractile amplitude (**C**), and uterine contractile irregularity (**E**) in two groups of mice. (**D**) Scatter plot showing the relationship between hotplate latency and contractile amplitude. The data are represented in mean ± SD. The comparison was made using Wilcoxon’s rank test. (**F**) Scatter plot showing the relationship between hotplate latency and contractile irregularity. In (D) and (F), each dot represents one data point. The dashed line is the regression line. Pearson’s correlation coefficient is shown. Symbols for statistical significance levels: NS: *P* > 0.05; **: *P* < 0.01; ***: *P* < 0.001.

While no difference in bodyweight was found between the two groups before the induction of adenomyosis and in 4, 8, and 12 weeks after induction (all 4 *P*-values ≥ 0.12; Fig. 5A), a multiple linear regression analysis incorporating the time of measurement and group identity (AM vs CTL) indicated a statistically significant time-dependent increase in bodyweight in both groups (*P* < 2.2 × 10^−16^) but mice with induced adenomyosis had lighter bodyweight as compared with control mice (*P* = 0.026, *R*^2^ = 0.70), possibly due to pain-suppressed food intake resulting from adenomyosis-induced pain.

While mice with induced adenomyosis had comparable hotplate latency with control mice before and 4 weeks after the induction (both *P*-values ≥ 0.10; Fig. 5B), they had significantly shorter latencies than those of control mice 8 and 12 weeks after induction (both *P*-values ≤ 0.007; Fig. 5B). For mice in the control group, the latency measured at 4, 8, and 12 weeks after the induction time did not change significantly as compared with the baseline levels (all 3 *P*-values ≥ 0.46). In contrast, the latency in mice with induced adenomyosis was progressively shortened, and was significantly shorter at 8 and 12 weeks (both *P*-values = 0.016) as compared with their baseline levels. Multiple linear regression incorporating time of measurement and group identity indicated that the group identity and time interaction was statistically significant (*P* = 4.8 × 10^−5^, *R*^2^=0.48), reflecting the divergent trend in latency between the two groups (Fig. 5B).

Consistent with the difference in latency, the difference in contractile amplitude between the two groups was comparable before the induction and 4 weeks after (both *P*-values ≥ 0.28) but became significant 8 and 12 weeks after the induction (both *P*-values ≤ 0.0013; Fig. 5C). In fact, the hotplate latency and contractile amplitude were negatively correlated (*r* = −0.59, *P* = 2.8 × 10^−7^; Fig. 5D).

We also calculated the uterine contractile irregularity and found that the difference in contractile irregularity was comparable 4 weeks after the induction (*P* = 0.13) but was significant before the induction (*P* = 0.038) and 8 and 12 weeks after the induction (both *P*-values ≤ 0.0047; Fig. 5E). The hotplate latency and contractile irregularity were negatively correlated (*r* = −0.52, *P* = 1.1 × 10^−5^; Fig. 5F).

**Figure 6.**
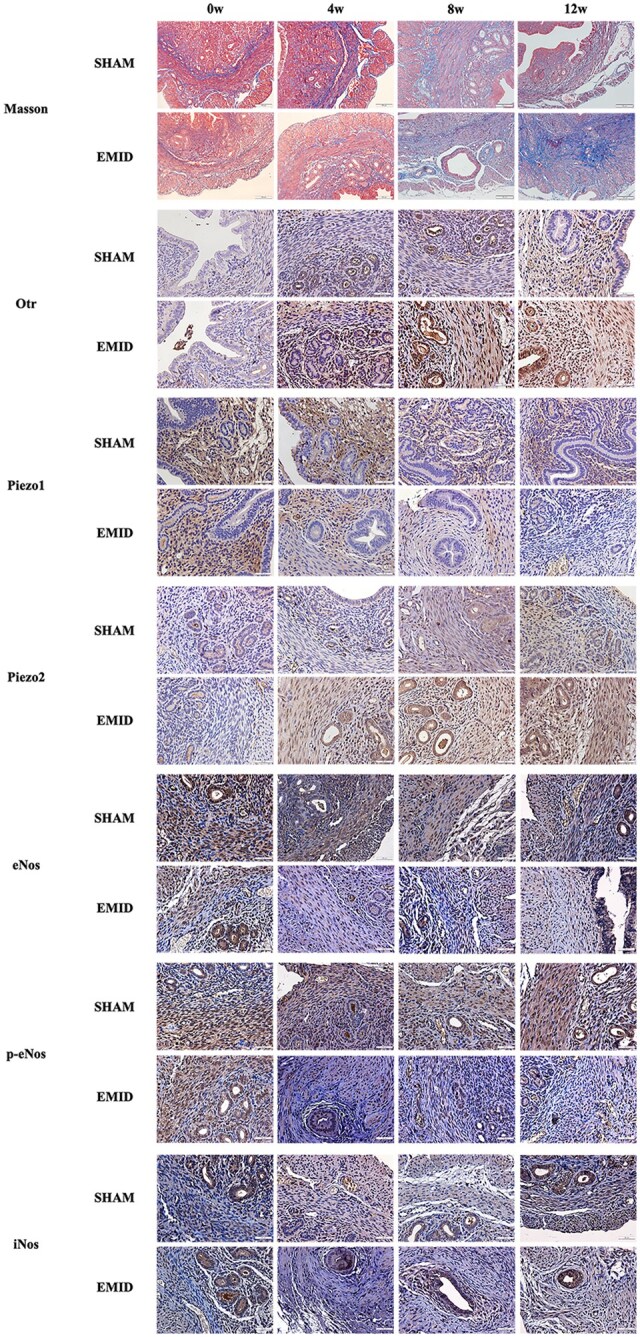
**Representative photomicrographs of immunohistochemistry and histochemistry**. Representative photomicrographs of immunohistochemistry of Otr, Piezo1, Piezo2, eNos, p-eNos, and iNos, along with the extent of fibrosis in myometrium neighboring adenomyotic lesions every 4 weeks after adenomyosis induction. Collagen fibers were stained blue, and muscle fibers were red with Masson trichrome staining. Immunoreactivity of Otr, Piezo1, Piezo2, eNos, p-eNos, and iNos was observed in both epithelial cells and myometrial cells, and Otr, Piezo1, and Piezo2 localized in the cell membrane. In contrast, eNos, p-eNos, and iNos localized both in the cell membrane and cytoplasm. Magnification: ×400. Scale bar, 50 μm. Otr, Oxytocin receptor, eNos, endothelial nitric oxide synthase; p-eNos, phospho-endothelial nitric oxide synthase; iNos, inducible nitric oxide synthase.

**Figure 7.**
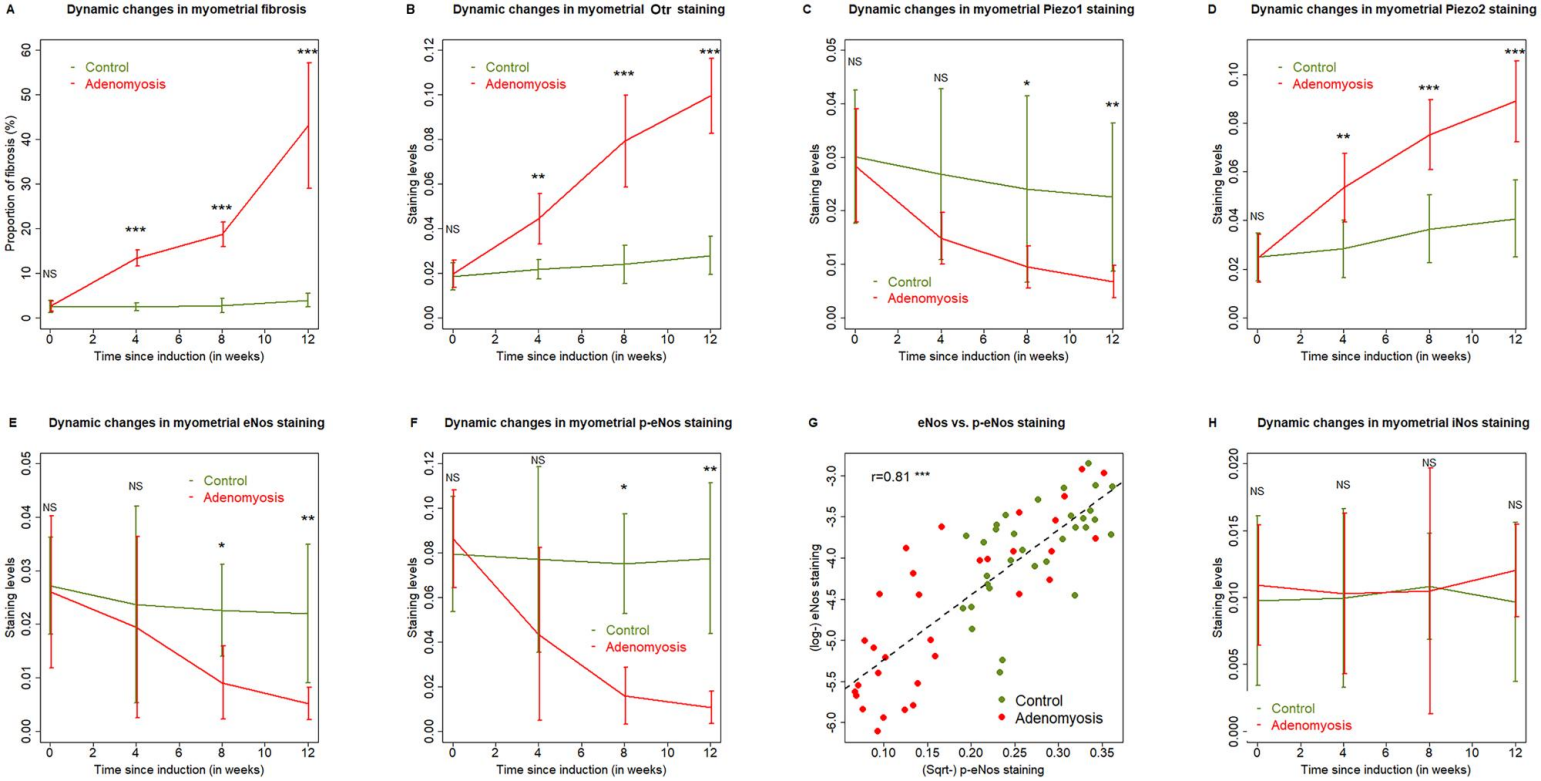
**Summary of mouse histochemistry and immunohistochemistry data.** Dynamic changes in the extent of myometrial fibrosis via Masson trichrome staining (**A**), Otr staining (**B**), Piezo1 staining (**C**), Piezo2 staining (**D**), eNos staining (**E**), p-eNos staining (**F**), and iNos staining (**H**) in two groups of mice. The data are represented in mean ± SD. The comparison was made using Wilcoxon’s rank test. (**G**) Scatter plot showing the relationship between eNos and p-eNos staining levels. The data are represented in mean ± SD. In (G), each dot represents one data point. The dashed line is the regression line. Pearson’s correlation coefficient is shown. Symbols for statistical significance levels: NS: *P* > 0.05; *: *P* < 0.05; **: *P* < 0.01; ***: *P* < 0.001.

Next, we performed an IHC analysis of Piezo1, Piezo2, and Otr and quantitated the extent of tissue fibrosis by Masson trichrome staining (Fig. 6). Piezo1, Piezo2, and Otr staining were observed mostly in the membrane of epithelium and myometrium. As expected, while there was no significant change in the extent of myometrial fibrosis during the entire experimental period (all 3 *P*-values ≥ 0.083), the extent of fibrosis in mice with induced adenomyosis increased linearly as the length of the induction time, but not the normal myometrium in control mice (*P* = 7.2 × 10^−15^, *R*^2^ = 0.87; Fig. 7A).

Similarly, the difference in myometrial staining of Otr between the two groups became statistically significant since 4 weeks after the induction (all 3 *P*-values ≤ 0.0027; Fig. 7B). In particular, while in control mice the myometrial Otr staining was virtually constant during the entire experimental period, in mice with induced adenomyosis its staining increased in a time-dependent fashion (*P* = 7.1 × 10^−13^, *R ^2^*= 0.82; Fig. 7B).

In contrast, while the myometrial staining of Piezo1 in control mice was constant during the entire experimental period, its staining in adenomyosis mice decreased in a time-dependent manner (*P* = 4.8 × 10^−8^, *R*^2^ = 0.64; Fig. 7C). As a result, the difference became statistically significant from 8 weeks after the induction (both *P*-values ≤ 0.046; Fig. 7C).

Consistent with the human data, the myometrial staining of Piezo2 in mice with induced adenomyosis was elevated in a time-dependent fashion (*P* = 8.9×10^−11^, *R*^2^= 0.76), and, compared with control mice, became significantly higher after 4 weeks following the induction (all 3 *P*-values ≤ 0.0047; Fig. 7D). In control mice, their staining levels were virtually stable, and only became significantly higher than the baseline level by the end of the experiment (*P* = 0.0498).

Similarly, myometrial staining of both eNos and p-eNos in control mice was constant over the entire experimental period (both *P*-values ≥ 0.29; Fig. 7E and F). In contrast, the staining levels of both were significantly decreased over time (both *P*-values ≤ 7.5 × 10^−5^, both *R*^2^’s ≥ 0.41). Thus, starting from the 8th week, mice with induced adenomyosis had significantly lower eNos and p-eNos staining levels as compared with controls (all *P*-values ≤ 0.010; Fig. 7E and F). The eNos staining levels correlated positively with those of p-eNos (*r* = 0.81, *P* = 4.4 × 10^−16^; Fig. 7G).

Consistent with the human data, no significant difference in the myometrial staining of iNos was found at all time points between the mice with and without induced adenomyosis (all *P*-values ≥ 0.13; Fig. 7H).

**Figure 8.**
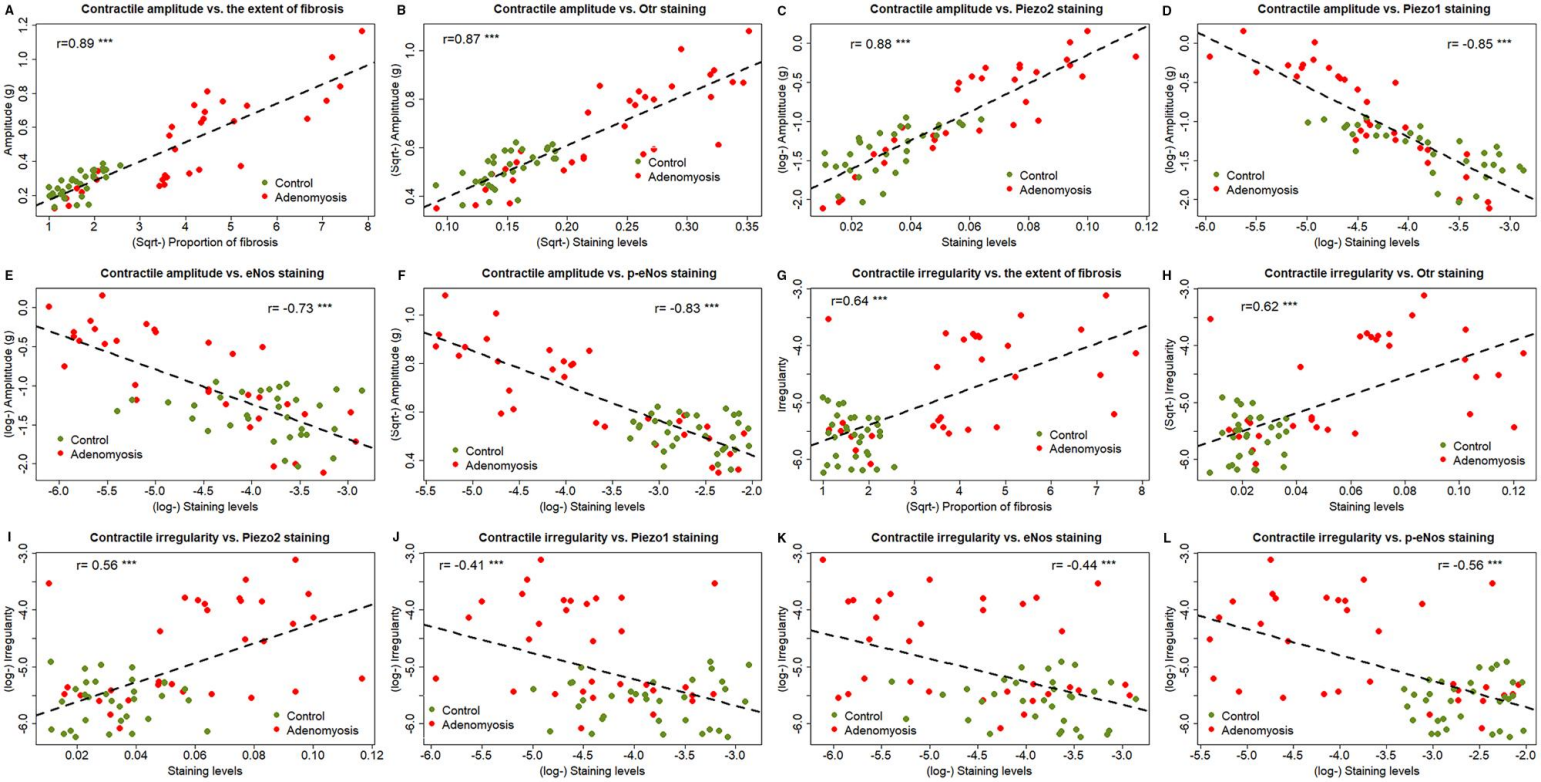
**Scatter plots showing the relationship between two variables.** Scatter plots showing the relationship between contractile amplitude and myometrial fibrosis (**A**), Otr staining levels (**B**), Piezo2 staining levels (**C**), Piezo1 staining levels (**D**), eNos staining levels (**E**), p-eNos staining levels (**F**), and between contractile irregularity and myometrial fibrosis (**G**), Otr staining levels (**H**), Piezo2 staining levels (**I**), Piezo1 staining levels (**J**), eNos staining levels (**K**), and p-eNos staining levels (**L**). In all plots, each dot represents one data point. The dashed line is the regression line. Pearson’s correlation coefficient is shown. Symbols for statistical significance levels: *: *P* < 0.05; **: *P* < 0.01; ***: *P* < 0.001.

The extent of myometrial fibrosis correlated positively with both Otr and Piezo2 staining (both *r*’s ≥ 0.92, both *P*-values < 2.2 × 10^−16^) but negatively with Piezo1 staining (*r*= −0.84, *P* < 2.2 × 10^−16^). Remarkably, both contractile amplitude and irregularity correlated positively with the extent of fibrosis (both *r*’s ≥ 0.64, *P* ≤ 1.4 × 10^−8^; Fig. 8A and G). Similarly, uterine contractile amplitude and irregularity correlated positively with the staining levels of Otr and Piezo2 (all *r*’s ≥ 0.56, all *P*-values ≤ 1.9 × 10^−6^; Fig. 8B, C, H, and I) but negatively with those of Piezo1, eNos, and p-eNos (all 6 *r*’s ≤ −0.41, all 6 *P*-values ≤ 0.0007; Fig. 8D–F and J–L).

As expected, the hotplate latency was found to be negatively correlated with the uterine contractile amplitude and irregularity (both *r*’s ≤ −0.52, *P* ≤ 1.1 × 10^−5^; Supplementary Fig. S3). It also correlated negatively with the extent of myometrial fibrosis, and immunostaining levels of Otr and Piezo2 (both *r*’s ≤ −0.52, *P* ≤ 1.1 × 10^−5^; Supplementary Fig. S3) but positively with that of Piezo1, eNos, and p-eNos (all 3 *r*’s ≥ 0.33, *P* ≤ 0.0073; Supplementary Fig. S3). No apparent relationship was found between the latency and myometrial iNos staining levels (*r* = 0.22, *P* = 0.08; Supplementary Fig. S3). Consistent with the human data, the staining levels of both eNos and p-eNos correlated positively with those of Piezo1 (both *r*’s ≥ 0.58, *P* ≤ 5.6 × 10^−7^) but negatively with those of Piezo2 (both *r*’s ≤ 0.67, *P* ≤ 1.9 × 10^−9^).

Multivariate linear regression on the hotplate latency with group identity (AM vs CTL), uterine contractile amplitude and irregularity, extent of myometrial fibrosis, and myometrial staining levels of Otr, Piezo2, Piezo1, eNos, p-eNos, and iNos as co-variates revealed that having adenomyosis, the contractile amplitude, and staining levels of Piezo1 and Otr are all negatively associated with the latency (all *P*-values ≤0.039, *R*^2^=0.53).

### Promoter hypermethylation in myometrial PIEZO1 in patients with adenomyosis

**Figure 9.**
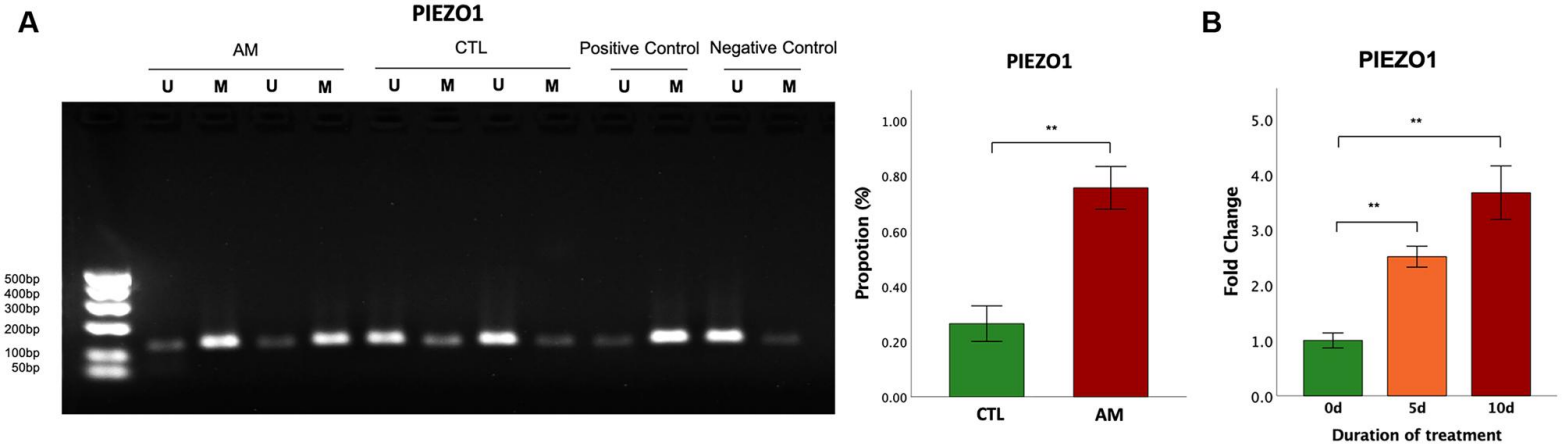
**Promoter hypermethylation in *PIEZO1* in adenomyotic lesions and its reactivation by HDAC inhibitor valproic acid (VPA).** (**A**) Left panel: Representative results of methylation-specific PCR of *PIEZO1* in myometrial cells derived from control myometrium (CTL) and adenomyosis (AM). U, reactions using the *PIEZO1* primer sets specific for the unmethylated CpG sites giving rise to a 130 bp band. M, reactions using the *PIEZO1* primer sets specific for the methylated CpG sites giving rise to a 131 bp band. Right panel: Bar plots showing the proportions of methylated samples in control myometrium (CTL) and adenomyosis (AM). N = 10 in each group. (**B**) Bar plots showing relative fold changes in gene expression levels of *PIEZO1* derived from myometrium neighboring adenomyotic lesions as a function of duration of treatment with VPA. N = 5 in each group. Symbols for statistical significance levels: **: *P* < 0.01.

Given the reduced lesional expression of PIEZO1 as compared with the control myometrium, we next investigated whether or not the promoter regions of *PIEZO1* gene in lesional myometrium were hypermethylated. We harvested myometrial tissue from both normal myometrium and myometrium around adenomyotic lesions, extracted their DNA, and performed an MSP analysis. We found that the promoter regions of *PIEZO1* were markedly hypermethylated in adenomyotic myometrium as compared with control myometrium (*P* = 0.0020; Fig. 9A).

To further demonstrate that *PIEZO1* is hypermethylated in adenomyosis, we treated uHSMCs derived from adenomyotic patients with valproic acid (VPA), a class I HDAC inhibitor. Treatment with VPA for 5 or 10 days significantly increased the expression levels of *PIEZO1* (both *P*-values ≤ 0.008; Fig. 9B) in uHSMCs. In particular, using linear regression incorporating the treatment duration as the single co-variable, we found that VPA treatment significantly elevated the gene expression levels of *PIEZO1* in a time-dependent manner (*R*^2^ = 0.79, *P* = 9.0 × 10^−6^; Fig. 9B).

## Discussion

We have demonstrated in this study that the myometrium surrounding adenomyotic lesions exhibits downregulation of PIEZO1 and eNOS alongside with upregulation of PIEZO2. We have further shown that PIEZO1 downregulation leads to downregulation of eNOS and subsequent reduced NO production, which may impair uterine relaxation. This could result in increased contractile amplitude and irregularity, thereby contributing to adenomyosis-associated dysmenorrhea. Notably, as the myometrium becomes more fibrotic and rigid, PIEZO1 expression is progressively suppressed while OTR expression is elevated. Finally, we identified promoter hypermethylation and consequent silencing of the *PIEZO1* gene in the myometrium proximal to adenomyotic lesions, which may underlie the increased uterine contractility and thus dysmenorrhea in women with adenomyosis.

Our findings align with previous reports of increased uterine contractility and OTR overexpression in the myometrium from women with adenomyosis (Mechsner *et al.*, 2010; Guo *et al.*, 2013; Zhang *et al.*, 2015; Orazov *et al.*, 2020), as well as with the observed downregulation of eNOS in the junctional zone (Liu *et al.*, 2013). They are consistent with studies indicating that PIEZO1 activation exerts negative inotropic effects on the myometrium, likely through the induction of PKA/AKT signaling, suggesting its potential role as tocolytic (Barnett *et al.*, 2023). Our data further agree with multiple reports demonstrating that PIEZO1 activation by shear stress is associated with eNOS activation (Li *et al.*, 2014a), vasodilation via NO (John *et al.*, 2018), and inhibition of human ureteral contractions via NO release (Liu *et al.*, 2024). Accumulating evidence indicates that PIEZO1-mediated Ca^2+^ influx can recruit PI3K/Akt and cAMP–PKA signaling cascades to activate eNOS and enhance NO synthesis. Upon mechanical or pharmacological activation, PIEZO1 has been shown to trigger eNOS phosphorylation and NO production via PI3K/Akt-dependent pathways and associated mechanosignalling complexes, resulting in vasodilation and flow-dependent vascular relaxation (Kuck *et al*., 2022). In parallel, cAMP effectors, including PKA, can stimulate eNOS through PI3K/Akt-mediated phosphorylation at Ser1177, further linking PKA activity to NO generation (García-Morales *et al*., 2017). More recently, direct PIEZO1 stimulation has been shown to markedly increase eNOS Ser1177 phosphorylation and NO release in red blood cells, reinforcing the functional coupling between PIEZO1 and eNOS (Sangha *et al.*, 2025). Thus, in contrast to the normal gravid uterus, myometrial PIEZO1 in adenomyosis is hypermethylated and downregulated, leading to reduced eNOS expression and NO production. This cascade may ultimately result in heightened uterine contractility and irregularity.

Aberrant uterine contractility has long been documented in women with adenomyosis (Mechsner *et al.*, 2010; Guo *et al.*, 2013) and also is implicated in endometriosis (Leyendecker *et al.*, 2002, 2004) and infertility (Bulletti and de Ziegler, 2006). However, its underlying mechanisms are still poorly understood. While aberrant expression of OTR, VP1αR (Akerlund, 2004; Mechsner *et al.*, 2010), PGF_2α_ (Jusuf *et al.*, 2024), and potassium channels (Shi *et al.*, 2016) may contribute to heightened contractility, impaired uterine relaxation has not been previously reported. Our data demonstrate that, as myometrial fibrosis and thus rigidity increases due to increased lesional fibrosis, PIEZO1 becomes hypermethylated and downregulated. This suppresses eNOS activation and reduces NO production, leading to increased uterine contractile amplitude and spasmodic contractions (irregularity) that contribute to dysmenorrhea. Furthermore, myometrial PIEZO1 downregulation may also facilitate axon regeneration (Song *et al.*, 2019; Li *et al.*, 2021), contributing to the increased nerve fiber density in myometrium observed in symptomatic adenomyosis (Zhang *et al.*, 2010).

Reduced NO promotes ECM deposition and exacerbates fibrosis by suppressing collagen degradation (Lundberg and Weitzberg, 2022). Evidence from renal (Chatziantoniou *et al.*, 1998), pulmonary (Hogaboam *et al.*, 1998), and dermal models (Dooley *et al.*, 2007) confirms that NO negatively regulates ECM accumulation across multiple fibrotic contexts. Our study demonstrates that increased ECM stiffness downregulates PIEZO1, fostering a pro-fibrotic feedback loop through enhanced ECM deposition. Moreover, increased myometrial rigidity elevates OTR expression, further promoting uterine contractility and irregularity (Mao *et al.*, 2011). While PIEZO2 suppression does not affect eNOS/NO, its overexpression is shown to be stiffness-dependent. Its precise role in adenomyosis requires further elucidation, but it may be involved in mechanosensing and mechanotransduction, potentially facilitating myometrial fibrogenesis (Freeberg *et al.*, 2025) or pain (Prato *et al.*, 2017; Xie *et al.*, 2025). Further investigation through PIEZO2 ablation may help elucidate its role in adenomyosis.

Our finding of differential expression of PIEZO1, PIEZO2, OTR, and eNOS/p-eNOS in myometrium proximal and distal to adenomyotic lesions indicates that the myometrium in adenomyosis is not entirely homogeneous. Instead, the transcriptome and proteome appear to vary depending on the proximity to lesions and consequent differential susceptibility to their influence. In fact, our observation of higher OTR immunostaining in the ipsilateral myometrium compared to the contralateral side is consistent with a previous report (Orazov *et al.*, 2020) and may help explain discrepant findings regarding myometrial eNOS expression in the literature (Liu *et al.*, 2013; Oh *et al.*, 2013).

The link we identified between PIEZO1 and eNOS, along with the discovery of *PIEZO1* hypermethylation, has important clinical implications. First, the stiffness-dependent increase in OTR and PIEZO2 expression, coupled with decreased PIEZO1 expression, underscores the progressive nature of adenomyosis. This is consistent with previous reports that the extent of lesional fibrosis correlates with the severity of dysmenorrhea (Liu *et al.*, 2018) and HMB (Huang *et al.*, 2022; Mao *et al.*, 2023, 2025). Second, myometrial PIEZO1/eNOS signaling appears to be a possible therapeutic target for adenomyosis-associated dysmenorrhea given its role in uterine hypercontractility and in light of the report that PIEZO1 is reported to inhibit axon regeneration via the NOS-protein kinase G pathway (Song *et al.*, 2019), which may account for hyperinnervation in adenomyosis (Zhang *et al.*, 2010). Third, while our study focused on adenomyosis-associated dysmenorrhea, the PIEZO1–eNOS aberration might also occur in other uterine disorders such as fibroids, and may also be a contributing factor for infertility resulting from embryo implantation failure because of adenomyosis or other pathologies. Lastly, given that VPA reactivates *PIEZO1* expression and subsequently induces eNOS, histone deacetylase inhibitors (HDACIs) appear to be promising therapeutics. This is particularly relevant since uterine contractility can be inhibited by HDACIs such as VPA and trichostatin A (TSA) (Moynihan *et al.*, 2008), and TSA can also suppress myometrial OTR expression (Guo *et al.*, 2013). Preclinical studies clearly demonstrate the potential of HDACIs in treating adenomyosis (Jichan *et al.*, 2010; Liu and Guo, 2011; Mao *et al.*, 2011; Guo *et al.*, 2013; Abdalla Ribeiro *et al.*, 2014; Nie and Liu, 2017a,b), and preliminary clinical studies have shown promise for VPA (Liu and Guo, 2008; Xishi *et al.*, 2010). In light of our finding of myometrial PIEZO1 hypermethylation, along with the hypermethylation of PR-B (Jichan *et al.*, 2010), Klotho, and PPARγ (Fan *et al.*, 2025) in adenomyosis, the use of HDACIs as a therapeutic intervention becomes even more compelling (Guo, 2025).

Adenomyosis frequently co-exists with endometriosis and uterine fibroids (Li *et al.*, 2014b; Leyendecker *et al.*, 2015). In our study, 30% and 13.3% of patients also had co-existing endometriosis and fibroids. Since both adenomyotic and endometriotic lesions progress to fibrosis (Guo, 2018) and since fibroids are fundamentally a fibrotic disease (Bhat *et al.*, 2023), all three diseases feature excessive ECM products. As such, these lesions, especially adenomyosis and fibroids, all lead to myometrial fibrosis and a stiff microenvironment, as well as myometrial downregulation of PIEZO1 and eNOS but upregulation of OTR and PIEZO2. All these lesions may also release profibrotic molecules, facilitating myometrial fibrosis. As shown in our study, the increased myometrial fibrosis leads to increased myometrial stiffness, which, in turn, results in downregulation of PIEZO1 and eNOS but upregulation of OTR and PIEZO2, leading to increased uterine contractility and irregularity. As such, we believe that our results are not affected by the co-morbidity with either endometriosis, fibroids, or both.

**Figure 10.**
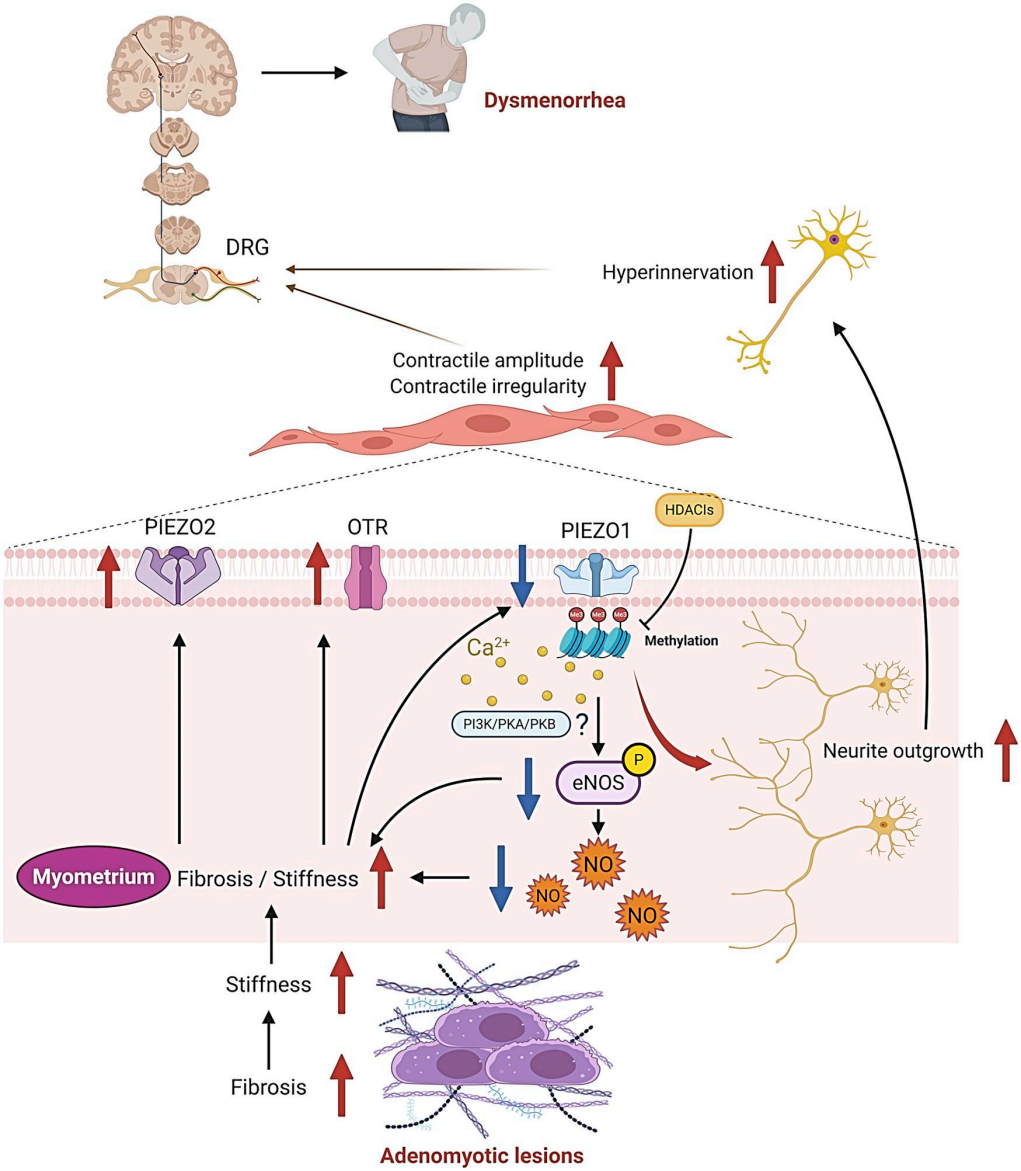
**Schematic illustration of the proposed mechanisms underlying adenomyosis-associated dysmenorrhea.** In adenomyosis, increased amplitude and irregularity of myometrial contractions, together with hyperinnervation, facilitate pain signaling via the dorsal root ganglia (DRG) to the central nervous system, contributing to perception of pain during menstruation (dysmenorrhea), and to central sensitization. Downregulation of *PIEZO1* expression, due likely to promoter hypermethylation, reduces Ca^2+^ influx, potentially through the PI3K/PKA or PKB pathways, leading to suppressed eNOS activation (through phosphorylation of Ser1177) and consequently decreased NO production. Loss of NO-mediated anti-fibrotic and myometrial-relaxing effects would promote extracellular matrix (ECM) deposition, inflammation, platelet aggregation, and heightened uterine contractions. PIEZO1 downregulation may further enhance neurite outgrowth, facilitating local hyperinnervation. Conversely, progressive fibrosis increases ECM stiffness, upregulates OTR, and further depresses PIEZO1, leading to reduced NO bioavailability and driving uterine hypercontractility. In addition, increased lesional fibrosis and myometrial fibrosis upregulate PIEZO2, which may further accelerate fibrogenesis. This establishes a self-sustaining vicious cycle that amplifies adenomyosis-related dysmenorrhea. DRG, dorsal root ganglia; OTR, oxytocin receptor; eNOS, endothelial nitric oxide synthase; NO, nitric oxide; Created in BioRender. Dingmin Yan. (2025) https://BioRender.com/ojglj3v.

In adenomyosis-associated dysmenorrhea, increased myometrial contractile amplitude (Guo *et al.*, 2013; Rees *et al.*, 2024) and irregularity (Rees *et al.*, 2024), coupled with hyperinnervation (Zhang *et al.*, 2010), transmit signals via the dorsal root ganglia to the central nervous system, contributing to pain and hyperalgesia. The downregulation of PIEZO1 expression might lead to reduced Ca^2+^ influx (Porto Ribeiro *et al.*, 2022), which inhibits phosphorylation of eNOS at Ser1177—potentially through the PI3K/PKA (Zhang and Hintze, 2006) or the PKB (Bir *et al.*, 2012) pathways—thereby decreasing NO production. As NO exerts anti-fibrotic effects and modulates smooth muscle relaxation (Dooley *et al.*, 2012), its reduction is likely to promote fibrogenesis (Kazakov *et al.*, 2013; Amador-Martinez *et al.*, 2019), inflammatory responses (Polte *et al.*, 2000; Cheng *et al.*, 2020), and platelet aggregation (Forstermann, 2010), driving uterine hypercontractility that exacerbates adenomyosis-associated dysmenorrhea. Furthermore, PIEZO1 downregulation may facilitate neurite outgrowth (Song *et al.*, 2019; Li *et al.*, 2021), resulting in hyperinnervation as seen in adenomyosis. Additionally, as lesion fibrosis progresses, concurrent ECM stiffness increases with reduced eNOS activity (Chung *et al.*, 2003; Yoshimura *et al.*, 2006) and NO levels (Chatziantoniou *et al.*, 1998; Dooley *et al.*, 2012), further promoting peri-lesional myometrial fibrosis. The increased stiffness of the fibrotic myometrium upregulates expression of OTR and PIEZO2, resulting in hypercontractility and further fibrosis. Thus, a vicious cycle is established that promotes lesional progression and exacerbates adenomyosis-associated dysmenorrhea (Fig. 10).

Our study has several strengths. First, from a mechanotransduction perspective, we investigated the possible involvement of PIEZO channels in adenomyosis and identified the crucial role of eNOS and NO production in mediating uterine contractility. Second, through a combination of IHC, quantification of gene and protein expression, MSP, and *in vitro* and *in vivo* experimentations using both human and mouse data, we have provided a series of interlocking evidence demonstrating PIEZO1 aberration in the adenomyotic myometrium and its contribution to dysmenorrhea. Lastly, by demonstrating that VPA can upregulate PIEZO1, we have provided additional support for the therapeutic potential of HDACIs (Guo, 2025).

Our study also has several limitations. First, while we demonstrated stiffness-dependent reduction of PIEZO1 but increase in PIEZO2 and OTR expression, the underlying mechanisms for these changes remain unelucidated. In particular, the relationship between PIEZO1 downregulation and augmented uterine contractility as well as exacerbated dysmenorrhea is technically an association and has not been directly demonstrated even though the link has been documented in other SMCs. Second, although we provided evidence that eNOS expression and NO production are determined by PIEZO1 expression levels, the precise mechanisms were not fully elucidated. These may be mediated by the PI3K/PKA or PKB (Zhang and Hintze, 2006; Bir *et al.*, 2012) pathways, but this would warrant future investigation. Third, while there is an excellent correlation between lesional stiffness and the extent of lesional fibrosis (Liu *et al.*, 2018) as well as concordance between reported endometrial fibrosis (Mao *et al.*, 2023) and endometrial stiffness as quantified by atomic force microscopy (Wang *et al.*, 2024), we did not specifically quantify the myometrial stiffness in Young’s modulus even though our *in vitro* experiments were performed with designated matrix stiffness (e.g. 5, 30, and 50 kPa). Still, given the reported average Young’s modulus of eutopic endometrium from patients with adenomyosis being 21.2 ± 1.6 kPa (vs 4.7 ± 0.4 kPa in control endometrium) (Wang *et al.*, 2024), we believe that our choice of the matrix stiffness is appropriate and reasonable. Future studies using atomic force microscopy should give us more precise measurements. Fourth, this study was conducted at a single institution, which might limit the generalizability of the findings to broader or more diverse populations. Although the patient population at our institution is rather homogeneous and the mechanobiological pathways examined (e.g. PIEZO1-related mechanisms) are highly conserved, validation in multi-center or more diverse populations would strengthen the external validity of our observations. Future studies incorporating samples from additional institutions and populations will be important to confirm the robustness and broader applicability of these results. Lastly, while we demonstrated the stiffness-dependent increase in PIEZO2 expression and that its suppression did not affect NO production, its precise role in adenomyosis has not been elaborated and requires more research.

To conclude, we have identified PIEZO1 downregulation, likely due to promoter hypermethylation, in myometrium from women with adenomyosis is responsible for attenuated eNOS activation and reduced NO production in myometrial SMCs. The resultant NO deficiency in myometrium may effectively confer inotropic effects, leading to increased contractile amplitude and irregularity, which in turn contributes to dysmenorrhea. Since HDACIs such as VPA can reactivate silenced PIEZO1, thereby activating eNOS and increasing NO production, they hold potential as therapeutics for adenomyosis.

## Supplementary Material

hoag013_Supplementary_Data

## Data Availability

The data presented in this study are available from the corresponding author upon written request specifying the purpose of obtaining the data.
