# Supplementary material for: Impaired PIEZO1 function drives uterine hypercontractility in adenomyosis-associated dysmenorrhea
Source: Hum Reprod Open. 2026 Feb 17;2026(2):hoag013. doi: 10.1093/hropen/hoag013 (PMC12981915; doi:10.1093/hropen/hoag013)
Supplement: hoag013_Supplementary_Data [file hoag013_supplementary_data.zip › HROPEN-25-0532.R2 Supp MM.docx]

**Impaired PIEZO1 Function Drives Uterine Hypercontractility in**

**Adenomyosis-Associated Dysmenorrhea**

**Dingmin Yan, Yuedong Wang, Xishi Liu, and Sun-Wei Guo**

**Supplementary Materials and Methods**

***Immunohistochemistry***

Tissue samples were fixed with 10% formalin (w/v) and paraffin embedded. Serial sections of 4 μm thickness were prepared from each paraffin block. The initial slide each series was stained with hematoxylin and eosin to verify the pathological diagnosis, while subsequent sections were used for immunohistochemical analysis of the following targets: PIEZO1 (1:400, PA5-72973; Thermo Fisher Scientific, Rockford, IL, USA), PIEZO2 (1:200, PA5-72976; Thermo Fisher Scientific), Oxytocin receptor (OTR) (1:50, cat No. 23045-1-AP, Proteintech Group Inc, Rosemont, IL, USA), phospho-endothelial nitric oxide synthase (Ser1177) (p-eNOS) (1:100, AF3247, Affinity, Cincinnati, OH, USA), endothelial nitric oxide synthase (eNOS) (1:100, AF0096, Affinity), inducible nitric oxide synthase (iNOS) (1:100, AF0199, Affinity). For negative controls, tissue samples were incubated with rabbit serum instead of primary antibodies.

Routine deparaffinization and rehydration of the sections were carried out following established protocols (Zhang et al., 2017). Antigen retrieval was performed using citrate buffer (pH 6.0, Shanghai Sun BioTech Company, Shanghai, China) for PIEZO1, PIEZO2, p-eNOS, eNOS and iNOS, while EDTA buffer (pH 9.0, Shanghai Sun BioTech Company) was used for OTR. Slides were heated in a pressure cooker containing boiling citrate buffer (pH 6.0) or EDTA buffer (pH 9.0) for 3 minutes until the pressure valve released, after which the cooker was filled with tap water to gently cool the slides. Following antigen retrieval, the slides were incubated with the primary antibodies overnight at 4°C. After rinsing, a horseradish peroxidase-labeled secondary antibody detection reagent (Sunpoly-HII; BioSun Technology Co, Ltd, Shanghai, China) was applied and incubated at room temperature for 30 minutes. Bound antibody complexes were visualized using diaminobenzidine staining, applied for 3 to 5 minutes or until optimal microscopic contrast was achieved. Slides were then counterstained with hematoxylin for 30 seconds and mounted. Images were acquired using an Olympus BX53 microscope (Olympus, Tokyo, Japan) equipped with an Olympus DP73 digital camera. For each sample, three to five randomly selected fields were captured at 400× magnification, and the mean optical density was quantified using Image Pro-Plus 6.0 software.

***Preparation of polyacrylamide gels***

Polyacrylamide gels with varying matrix stiffness values were prepared following a previously described protocol (Fischer et al., 2012), with minor modifications as implemented in our previous work (Huang et al., 2022). Briefly, glass coverslips of 24 mm in‐diameter (Thermo Fisher Scientific, Waltham, MA, USA) were thoroughly cleaned, sterilized, and dried. They were then soaked with a 0.5% solution of 3‐aminopropyltriethoxysilane (Sigma‐Aldrich, St. Louis, MO, USA) to promote covalent attachment of the polyacrylamide gels, followed by activation with 0.5% glutaraldehyde (Sigma‐Aldrich). The processed coverslips were stored in a light-protected environment until further use. To prepare polyacrylamide gels with target stiffness values of 5, 30, and 50 kPa, a mixture was formulated using 40% acrylamide solution (Bio‐Rad Laboratories, Hercules, CA, USA), 2% bis‐acrylamide solution (Bio‐Rad), and sterilized double‐distilled H2O in specific ratios. A volume of 400 μL of this mixture was pipetted onto a hydrophobic surface formed by parafilm (Bemis Company, Neenah, WI, USA). An activated glass coverslip was carefully placed over the droplet and allowed to polymerize for 30 minutes. For extracellular matrix (ECM) protein conjugation, the polymerized gel surface was activated with Sulfo‐SANPAN solution (1 mg/ml; Thermo Scientific) and immediately exposed to ultraviolet light (at 365 nm) for 15 min to facilitate crosslinking. Afterward, the gel surface was coated with type I collagen (0.2 mg/ml; BD Biosciences, Franklin Lake, NJ, USA) and incubated overnight at 4℃. The following day, the gels were acclimatized at room temperature for one hour, and then soaked in DMEM medium to go through hydration, equilibration, and sterilization under ultraviolet light for 4 hours.

***Western blot analysis***

For cellular protein extraction, uHSMCs subjected to different treatments for 72 hours were lysed using Radio-Immunoprecipitation Assay (RIPA) buffer (Fermentas, Thermo Fisher Scientific, Pittsburgh, PA, USA). For tissue protein extraction, smooth muscle tissue around the lesion from patients with adenomyosis were homogenized. The tissues were rinsed in pre-cooled PBS to completely remove residual blood, and 100 mg of tissue was minced and homogenized in 1 mL of RIPA buffer with a magnetic bead homogenizer. Protein concentration was determined using a bicinchoninic acid (BCA) protein quantitative analysis kit (P0010S, Beyotime, Shanghai, China). Protein samples were separated by electrophoresis on 10% sodium dodecyl sulfate–polyacrylamide gels (SDS-PAGE), and transferred onto polyvinyl difluoride (PVDF) membranes (Bio-Rad, Hercules, CA, USA). The membranes were incubated overnight at 4 °C with the following primary antibodies: PIEZO1 (1:500, PA5-72973; Thermo Fisher Scientific), PIEZO2 (1:500, PA5-72976; Thermo Fisher Scientific), OTR (1:1000, cat No. 23045-1-AP, Proteintech Group Inc, IL, USA), p-eNOS (1:1000, AF3247, Affinity), eNOS (1:1000, AF0096, Affinity), iNOS (1:1000, AF0199, Affinity), and glyceraldehyde-3-phosphate dehydrogenase (GAPDH; 1:1000; 5174; CST, Boston, MA, USA). Following incubation with horseradish peroxidase-conjugated secondary antibodies at room temperature for one hour, protein bands were visualized using enhanced chemiluminescence (ECL) reagents (Pierce, Thermo Scientific, Rockford, IL, USA) and then digitized on Image Quant LAS 4000 mini (GE Healthcare). Image quantification was carried out with Quantity One software (Bio-Rad).

**References**

Fischer RS, Myers KA, Gardel ML, Waterman CM. Stiffness-controlled three-dimensional extracellular matrices for high-resolution imaging of cell behavior. *Nat Protoc* 2012;7: 2056-2066.

Huang Q, Liu X, Guo SW. Higher fibrotic content of endometriotic lesions is associated with diminished prostaglandin E2 signaling. *Reprod Med Biol* 2022;21: e12423.

Zhang Q, Liu X, Guo SW. Progressive development of endometriosis and its hindrance by anti-platelet treatment in mice with induced endometriosis. *Reproductive biomedicine online* 2017;34: 124-136.
