# Supplementary figures and images for: Impaired PIEZO1 function drives uterine hypercontractility in adenomyosis-associated dysmenorrhea
Source: Hum Reprod Open. 2026 Feb 17;2026(2):hoag013. doi: 10.1093/hropen/hoag013 (PMC12981915; doi:10.1093/hropen/hoag013)

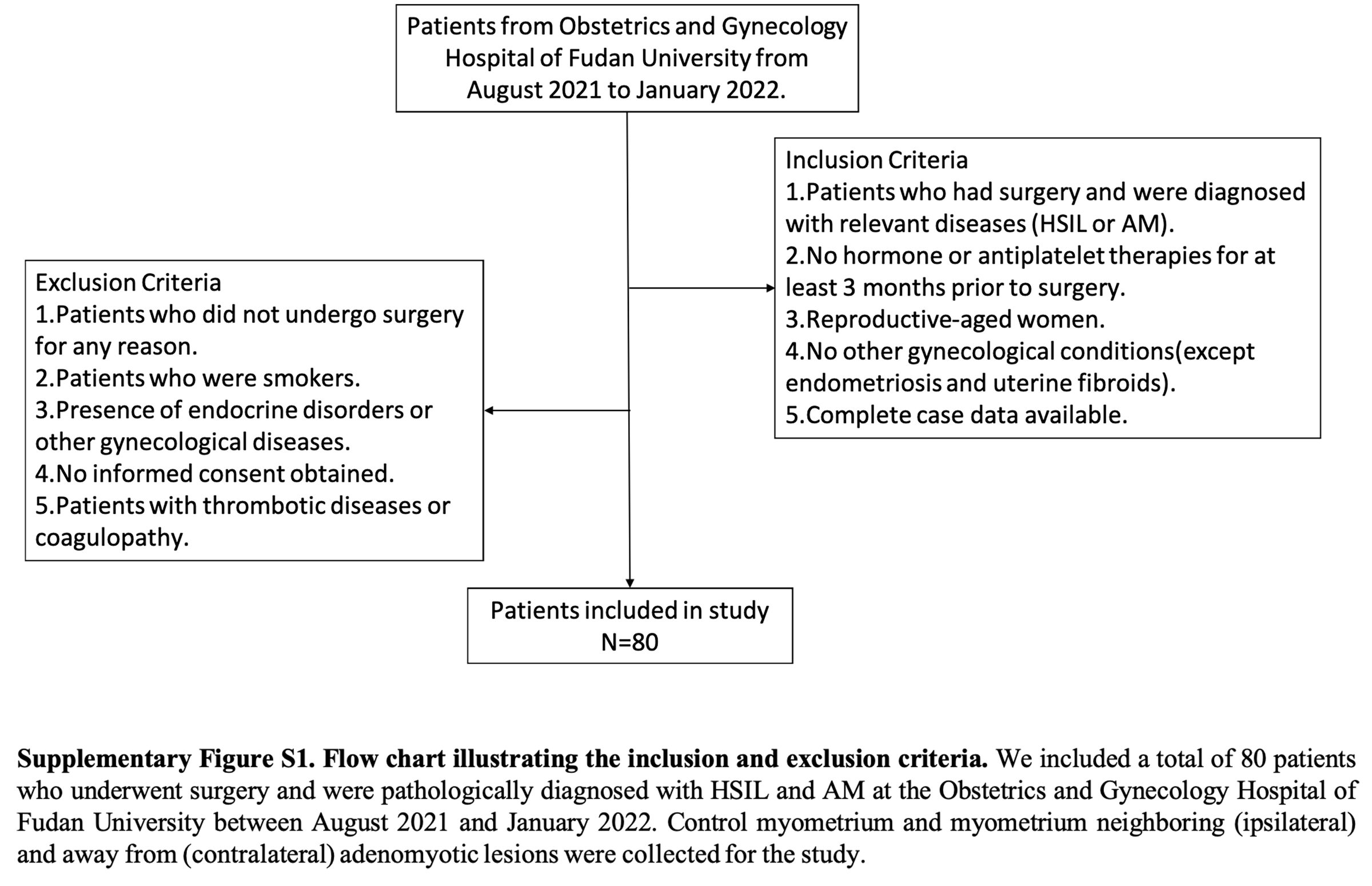

Supplement: hoag013_Supplementary_Data [file hoag013_supplementary_data.zip › Figure S1.tif]

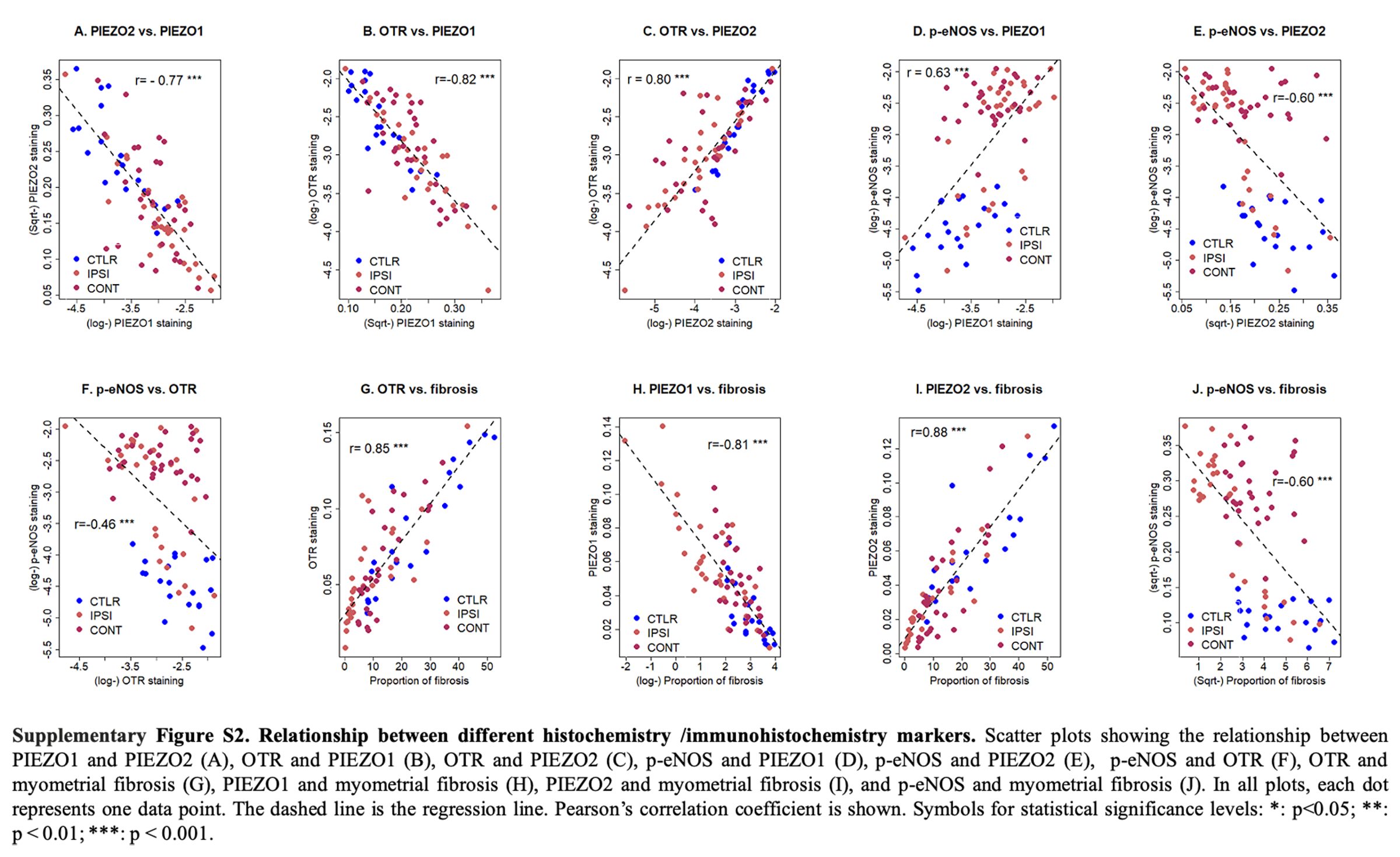

Supplement: hoag013_Supplementary_Data [file hoag013_supplementary_data.zip › Figure S2.tif]

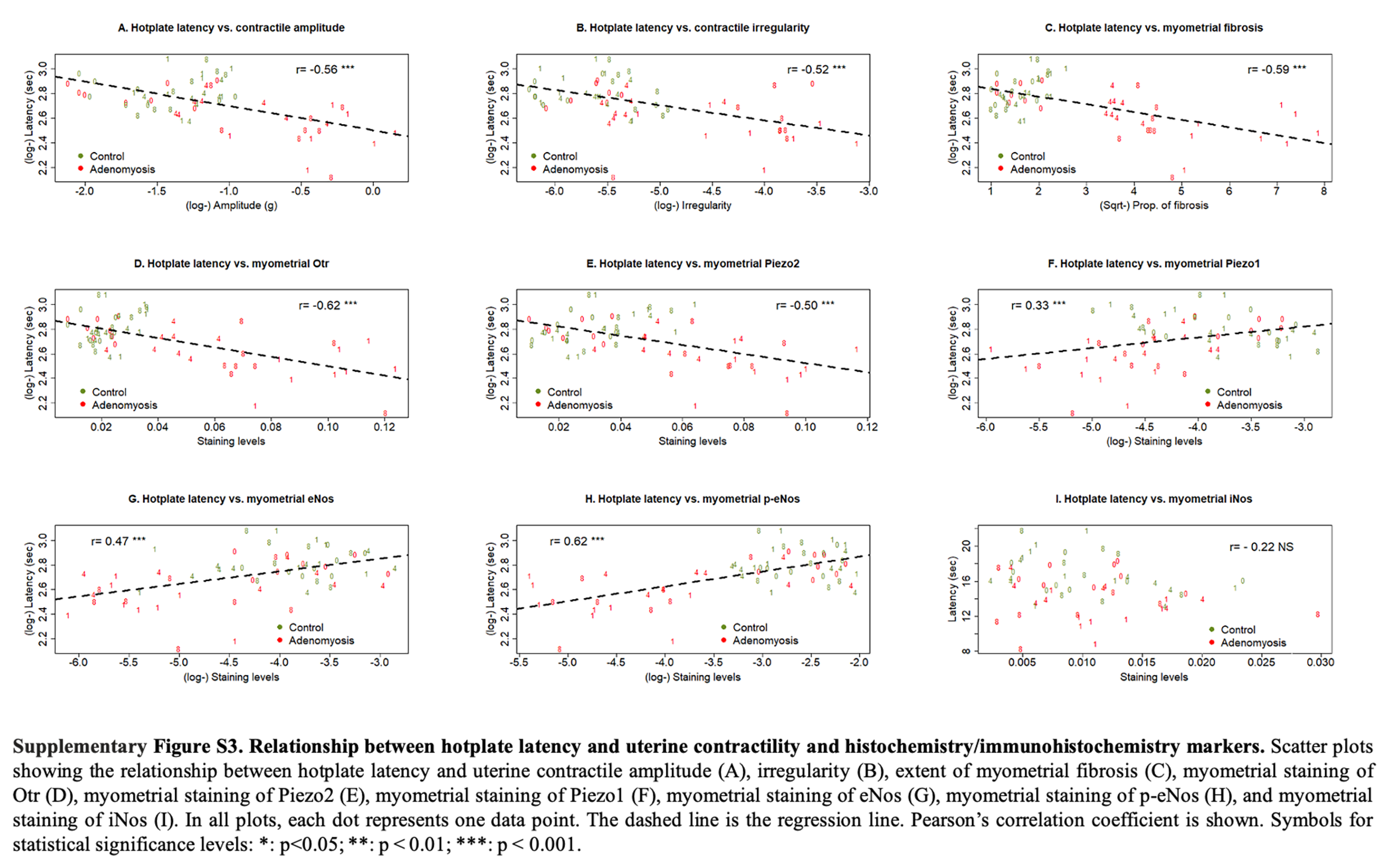

Supplement: hoag013_Supplementary_Data [file hoag013_supplementary_data.zip › Figure S3.tif]
